# Pitfalls of the most commonly used models of context dependent substitution

**DOI:** 10.1186/1745-6150-3-52

**Published:** 2008-12-16

**Authors:** Helen Lindsay, Von Bing Yap, Hua Ying, Gavin A Huttley

**Affiliations:** 1 Computational Genomics Laboratory, John Curtin School of Medical Research, The Australian National University, Canberra, ACT 0200, Australia; 2 Department of Statistics and Applied Probability, National University of Singapore, Kent Ridge, Singapore

## Abstract

**Background:**

Neighboring nucleotides exert a striking influence on mutation, with the hypermutability of CpG dinucleotides in many genomes being an exemplar. Among the approaches employed to measure the relative importance of sequence neighbors on molecular evolution have been continuous-time Markov process models for substitutions that treat sequences as a series of independent tuples. The most widely used examples are the codon substitution models. We evaluated the suitability of derivatives of the nucleotide frequency weighted (hereafter NF) and tuple frequency weighted (hereafter TF) models for measuring sequence context dependent substitution. Critical properties we address are their relationships to an independent nucleotide process and the robustness of parameter estimation to changes in sequence composition. We then consider the impact on inference concerning dinucleotide substitution processes from application of these two forms to intron sequence alignments from primates.

**Results:**

We prove that the NF form always nests the independent nucleotide process and that this is not true for the TF form. As a consequence, using TF to study context effects can be misleading, which is shown by both theoretical calculations and simulations. We describe a simple example where a context parameter estimated under TF is confounded with composition terms unless all sequence states are equi-frequent. We illustrate this for the dinucleotide case by simulation under a nucleotide model, showing that the TF form identifies a CpG effect when none exists. Our analysis of primate introns revealed that the effect of nucleotide neighbors is over-estimated under TF compared with NF. Parameter estimates for a number of contexts are also strikingly discordant between the two model forms.

**Conclusion:**

Our results establish that the NF form should be used for analysis of independent-tuple context dependent processes. Although neighboring effects in general are still important, prominent influences such as the elevated CpG transversion rate previously identified using the TF form are an artifact. Our results further suggest as few as 5 parameters may account for ~85% of neighboring nucleotide influence.

**Reviewers:**

This article was reviewed by Dr Rob Knight, Dr Josh Cherry (nominated by Dr David Lipman) and Dr Stephen Altschul (nominated by Dr David Lipman).

## Background

Sequence neighborhoods have been identified as exerting a strong influence on mutation with the most striking illustration being the elevated mutation rate affecting C within the dinucleotide CpG. This elevated mutation rate putatively arises because C within the CpG dinucleotide is the preferred target for modification by DNA methylases [1] and the resulting modified base, 5-methyl-cytosine (henceforth 5 mC), has an elevated mutation rate [2-4]. Analyses of sequence neighborhoods of human mutations have established, for this species at least, that a mutagenic influence of neighboring nucleotides on mutation rates is not restricted to CpG dinucleotides [5]. As different mutagenic and repair processes target different classes of sequence, determining how the substitution rate of a nucleotide is affected by the identity of neighboring nucleotides can assist in identifying the metabolic origins of a substitution. Establishing the relative importance of neighboring nucleotides and the etiology of these effects therefore has important implications for relating specific metabolic processes to existing genetic variation [6,7]. The contributions of factors that affect mutation rates can be identified using analyses of substitution rates. In the absence of natural selection, mutation and substitution rates are equal [8], allowing mutation rates to be inferred from neutral substitution rates. Since variation in substitution rates among sequence residues strongly influences estimation of evolutionary relationships [9-11], improved understanding of the origins of these effects, and how to correctly model them, will further benefit the reconstruction of accurate phylogenies.

Estimation of neighborhood effects from comparative genomic sequence data has been performed using so-called context dependent substitution models. A number of approaches have been developed which can be broadly classified according to whether they treat sequences as a series of independent tuples [12-14] or not [15-17]. We restrict our attention here to the independent tuple model class. All subsequent statements concerning context dependent models refer to the independent tuple case. Early models of context dependent substitution focused on measuring effects arising from RNA secondary structure [18] or from the genetic code in protein coding sequences [19,20]. (While employed for analysis of non-neighboring nucleotides involved in RNA stem structures, the model of Schöninger and von Haeseler [[18], hereafter SvH] is also a context dependent model.) The codon models constitute a special case of independent tuple models where sequence states that correspond to stop codons are excluded from the state-space, e.g. a codon model for the standard genetic code has 61 states corresponding to the sense codons, 3 states less than the full trinucleotide state-space of 64. While the originally defined codon models of Muse and Gaut [[19], hereafter MG] and Goldman and Yang [[20], hereafter GY] differed considerably in how they represented the influence of natural selection, with the model of GY in particular seeking to employ aspects of amino-acid chemistry, subsequent refinements [21] have resulted in the MG and GY models being more similar with respect to their exchangeability parameters.

The primary difference between these model forms of interest here is their different weighting of exchanges in the instantaneous rate matrix. In the MG codon model, the rate of nucleotide substitution within a codon context is weighted by the resulting nucleotide frequency. In the SvH and GY models, the rates are proportional to the frequency of the resulting tuple (doublet or codon respectively). We therefore classify models according to whether rates are weighted by nucleotide frequencies (hereafter NF models) or tuple frequencies (hereafter TF models). The effect of these differing definitions is on the expected equilibrium frequencies of tuples, with those under NF being the product of the independent monomer frequencies [22] (for the codon case, these are normalized for omission of the stop codons). The TF model formulation has proved more popular and several studies of context dependent substitution in non-coding sequences have used derivatives of the TF rate matrix form. Of particular interest has been assessment of the contribution of specific processes such as methylation to the overall rate of substitution [13,16,23]. A broader examination of the properties of a series of fully parameterized models both for independent and overlapping-tuple cases has also been done [16], demonstrating a substantial improvement in fit conferred by context dependent models over independent nucleotide models.

The distinct equilibrium frequencies expected under the TF and NF models indicate they differ in their relationship to an independent monomer process. Previous efforts at understanding the distinct properties of these model forms were performed on the more complex case of understanding protein coding sequence evolution and were based on contrasting statistical properties derived from analysis of codon alignments simulated under a TF variant [24], an approach acknowledged by the original authors as biased towards the TF form. The exact nature and significance of the difference between these model classes for general independent tuple models remains to be explored.

We suggest that a context dependent substitution model should specify the null model of context independence with fewer parameters than required to specify alternate hypotheses of context dependence. Here we show that the NF form satisfies this condition, that aside from very special cases the TF form does not and that naïve use of TF leads to incorrect detection of context effects. In light of the latter finding, we consider the consistency of the two model forms when applied to real data. Specifically, we reconsider the importance of nearest neighbor sequence context using reversible, stationary and homogeneous models of dinucleotide substitution.

## Results

### The relationship of NF and TF to an independent nucleotide process

We present the NF and TF models for dinucleotides but note that essentially the same treatment applies to trinucleotides and larger units. We also note here that different model forms will be referenced using the following notation: NF/TF refers to the component of a substitution rate matrix that stems from the motif probabilities while the subscript (e.g. nuGTR, explained below) refers to the exchangeability terms (*r*).

Let *q *denote a general time-reversible (GTR) substitution rate matrix [25,26] on nucleotides, i.e., for *i *≠ *j*, with elements *q*(*i*, *j*) = *r*(*i*, *j*) *π *(*j*) for some symmetric matrix *r *and equilibrium distribution frequencies *π*. For *t *≥ 0, let *p*_*t *_be the transition matrix across a time interval of length *t*. The element *p*_*t*_(*i*, *j*) is the conditional probability that the state at time *t *is *j *given that it is *i *at time 0. We have

(1) $$\underset{t\to 0}{\lim}p_{t}=I,$$

where *I *is the identity matrix. Furthermore, *q *is the derivative of *p*_*t *_at *t *= 0:

(2) $$\underset{t\to 0}{\lim}\frac{p_{t}-I}{t}=q$$

View two independent nucleotides undergoing the process defined by *q *as a unit, so that we have a reversible process on the dinucleotides with transition matrix *P*_*t *_and rate matrix *Q*. We now derive an expression for *Q*. By (2), for distinct dinucleotides *a *= *i*_1_*i*_2 _and *b *= *j*_1_*j*_2_,

$$\begin{array}{c} Q(a,b)=\underset{t\to 0}{\lim}\frac{P_{t}(a,b)}{t}\\ =\underset{t\to 0}{\lim}\frac{p_{t}(i_{1},j_{1})p_{t}(i_{2},j_{2})}{t} \end{array}$$

There are two cases to consider. (i) *a *and *b *differ in both positions. By (1) and (2),

$$Q(a,b)=\underset{t\to 0}{\lim}\frac{p_{t}(i_{1},j_{1})}{t}\times \underset{t\to 0}{\lim}p_{t}(i_{2},j_{2})=q(i_{1},j_{1})\times 0=0.$$

(ii) *a *and *b *differ in exactly one position. In the case where they differ at the first position, the same calculation gives

$$Q(a,b)=\underset{t\to 0}{\lim}\frac{p_{t}(i_{1},j_{1})}{t}\times \underset{t\to 0}{\lim}p_{t}(i_{2},j_{2})=q(i_{1},j_{1})\times 1=q(i_{1},j_{1}).$$

The calculation for the second position case is analogous. Thus,

$$Q(a,b)=\{\begin{array}{ll} 0 & i_{1}\neq j_{1},i_{2}\neq j_{2}\\ r(i_{1},j_{1})\pi (j_{1}) & i_{1}\neq j_{1},i_{2}=j_{2}\\ r(i_{2},j_{2})\pi (j_{2}) & i_{1}=j_{1},i_{2}\neq j_{2} \end{array}$$

Now we generalise by defining a dinucleotide rate matrix as follows,

$$Q_{\text{NF}}(a,b)=\{\begin{array}{ll} 0 & i_{1}\neq j_{1},i_{2}\neq j_{2}\\ r_{\text{NF}}(a,b)\pi (j_{1}) & i_{1}\neq j_{1},i_{2}=j_{2}\\ r_{\text{NF}}(a,b)\pi (j_{2}) & i_{1}=j_{1},i_{2}\neq j_{2} \end{array}$$

where the 16 × 16 *r*_NF _is symmetric. This new process is an NF (nucleotide frequency weighted) model. It will be shown in the next paragraph that its equilibrium frequencies are homogeneous multiplicative, i.e., *π *(*a*) = *π *(*i*_1_) *π *(*i*_2_), and that it is reversible. (As an aside, we note that it is possible to generalize the definition of NF to allow for position-specific differences in *π*.) Within NF, the nucleotide GTR process is an appropriate null model for investigating context effects. The joint contribution of various specific contexts can be easily specified and estimated from data by exploiting a whole spectrum of intermediate models. For this reason, the baseline nucleotide GTR process is called NF_nuGTR_.

The more widely used TF models are specified in a slightly, but crucially, different way from the above. In a TF (tuple frequency weighted) dinucleotide rate matrix, if *a *≠ *b*, then

$$Q_{\text{TF}}(a,b)=\{\begin{array}{ll} 0 & i_{1}\neq j_{1},i_{2}\neq j_{2}\\ r_{\text{TF}}(a,b)\pi (b) & \text{otherwise} \end{array}$$

where *r*_TF _is symmetric and *π *are the equilibrium frequencies. As *π *is not necessarily multiplicative, then the TF form is the most general reversible rate matrix for dinucleotides in the class of models where each substitution event involves only one nucleotide. The TF models with multiplicative *π *are precisely the NF models. To see this, first note that every NF model can be written in TF form by letting *π *be multiplicative. Then, for *a *and *b *differing in exactly one position,

$$r_{\text{TF}}(a,b)=\{\begin{array}{ll} r_{\text{NF}}(a,b)/\pi (j_{2}) & i_{1}\neq j_{1},i_{2}=j_{2}\\ r_{\text{NF}}(a,b)/\pi (j_{1}) & i_{1}=j_{1},i_{2}\neq j_{2} \end{array}$$

Conversely, if in a TF model *π *is multiplicative, then using the above relationship gives the same model in NF form. As a consequence, NF models have multiplicative equilibrium frequencies and are reversible. As for NF, the TF model where the *r*_TF _terms depend on the corresponding nucleotide substitution and nothing else is called TF_nuGTR_. When the *r*_TF _terms are symmetric and otherwise unconstrained, the model is called TF_diGTR_.

The fact that TF_diGTR _is a general reversible process makes it intuitive to expect it to be the "right" generalization of the nucleotide GTR process, i.e., that TF_nuGTR _with multiplicative equilibrium frequencies is the nucleotide GTR process, but this is false unless the nucleotides are equally frequent. For example, *q*_TF_(AA, AC) = *q*_TF_(CA, CC) implies *r*_TF_(AA, AC) *π *(A) = *r*_TF_(CA, CC) *π *(C), so that the two *r*_TF _terms are equal only if *π *(A) = *π *(C). This property predisposes TF to misinterpretations. Suppose that data are generated from the nucleotide GTR process, or equivalently, NF_nuGTR_, with unequal nucleotide frequencies. The maximized log likelihood under TF_nuGTR _will be appreciably less than that under NF_nuGTR_, which will result in a large gain in maximized log likelihood when TF_nuGTR _is compared with TF_diGTR _(which contains the nucleotide GTR process), leading to a spurious detection of context effects; but the NF models will behave as expected. We demonstrated this by simulating 100 50 kbp long alignments under NF_nuGTR_, using parameters estimated from the genomic alignment of orthologous intron sequences from human, chimpanzee and macaque for ENSG00000003147. The likelihood ratio statistic LR between NF_nuGTR _and NF_diGTR_, i.e., twice the difference in the maximized log likelihoods, has an approximate *χ*^2 ^distribution with 42 degrees of freedom, as predicted by theory; in particular, their average and standard deviation (SD) were 42.2 and 9.2. However, between TF_nuGTR _and TF_diGTR _the LR was typically very large, averaging to 161.0 with an SD of 25.1.

The TF form can also be unsatisfactory for detecting real context effects. Suppose that data are generated from TF_nuGTR _with non-multiplicative *π*, which is not a nucleotide GTR model. Then the LR between TF_nuGTR _and TF_diGTR _can be so small as to obscure the context effects. Somewhat unexpectedly, comparing NF_nuGTR _to NF_diGTR _can still succeed. This is illustrated by a similar simulation as in the previous paragraph, except that the data come from TF_nuGTR_. The TF_diGTR _LR averaged to 43.3 with an SD of 10.1, like a *χ*^2 ^distribution with 42 degrees of freedom, but for NF_diGTR _the average and SD were 313.9 and 30.4.

### Parameters estimated under a TF form can be misleading

We now show more clearly how TF can be misleading. Let *π *be a probability distribution on states {R, Y}. Consider an analogue of NF on {RR, RY, YR, YY} with rate matrix

$$Q_{\text{NF}}(\kappa )=\left[\begin{array}{cccc} - & \pi (\text{Y}) & \pi (\text{Y}) & 0\\ \pi (\text{R}) & - & 0 & \kappa \pi (\text{Y})\\ \pi (\text{R}) & 0 & - & \kappa \pi (\text{Y})\\ 0 & \kappa \pi (\text{R}) & \kappa \pi (\text{R}) & - \end{array}\right]$$

The diagonal entries are omitted since they are determined by the off-diagonal entries. *κ *measures the effect of a neighboring R on substitution rates, with *κ *= 1 corresponding to no context effect, analogous to a nucleotide process. Let a long pairwise alignment be generated by this process. Since it is nested in TF, fitting TF to the data will give *Q*_NF_(*κ*) up to a constant multiple, but in this form

$$Q_{\text{TF}}(K)=\left[\begin{array}{cccc} - & \pi (\text{R})\pi (\text{Y}) & \pi (\text{Y})\pi (\text{R}) & 0\\ \pi (\text{R})\pi (\text{R}) & - & 0 & K\pi (\text{Y})\pi (\text{Y})\\ \pi (\text{R})\pi (\text{R}) & 0 & - & K\pi (\text{Y})\pi (\text{Y})\\ 0 & K\pi (\text{R})\pi (\text{Y}) & K\pi (\text{Y})\pi (\text{R}) & - \end{array}\right]$$

where

(3) K=κπ(R)/π(Y)

If *κ *= 1, then the MLE of *κ *will be close to 1 by the consistency property. However, the MLE of K will be close to *π *(R)/*π *(Y), an erroneous detection of a context effect if *π *(R) ≠ *π *(Y). More generally, K and *κ *will be consistent if *π *(R) = *π *(Y), but K will be larger or smaller according to whether *π *(R) > *π *(Y) or *π *(R) <*π *(Y).

**Figure 1:**
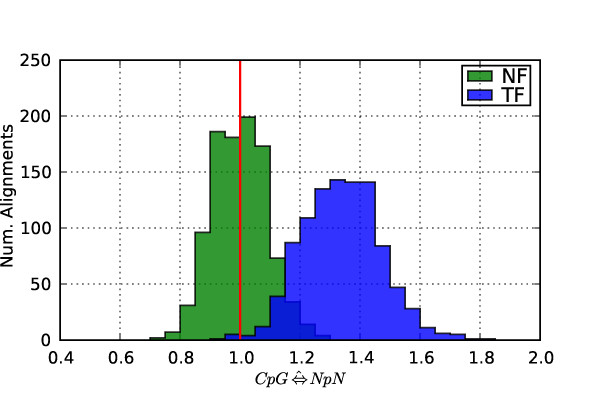
**Context effects are detected using TF when none exist**. Sequences were simulated under a nucleotide GTR model with *π *(*A*) + *π *(*T*) ≈ 0.6. A single dinucleotide parameter for *CG *⇔ *NN *was added to the TF_nuGTR _and NF_nuGTR _dinucleotide baseline models. The red vertical line represents the expected value under the null hypothesis (the parameter has no effect). x-axis is the MLE of *CG *⇔ *NN*, y-axis is the number of simulated alignments.

We illustrate the impact of this confounding of exchangeability parameters in TF with nucleotide composition by estimation of a context parameter from simulated data. We simulated 1000 alignments under NF_nuGTR _using the same parameter estimates as for the previous simulation (see Methods for details). For each alignment we fit variants of the NF_nuGTR _and TF_nuGTR _models that had been extended to include a single additional rate term for CpG substitutions (*CG *⇔ *NN*). Frequency histograms of the resulting maximum-likelihood estimates (MLEs) of the context parameter ($CG\hat{\Leftrightarrow }NN$) obtained under each model form are presented in Figure 1. As the alignments were simulated without any sequence context effects on CpG substitution, the null hypothesis of context independence is true and the expected value of *CG *⇔ *NN *is therefore 1. The $CG\hat{\Leftrightarrow }NN$ distribution obtained against the NF_nuGTR _baseline spans this expected value, whilst the distribution of $CG\hat{\Leftrightarrow }NN$ estimated under TF_nuGTR _is strongly right-shifted. This result is consistent with the proofs above showing the relationship of NF model forms to an independent nucleotide process, and the confounding (relative to this process) of exchangeability parameters under TF.

### The fit of TF and NF to primate intron alignments differs substantially

The analytical results indicate that the TF and NF forms will exhibit distinct model likelihoods and parameter MLEs – the critical statistics used for drawing inference on the importance of parameters and the nature of their effects. We contrasted the practical impact of the differences in the two model forms by analysis of aligned orthologous intron sequences from the human, chimpanzee and macaque primate lineages. Intronic sequences were used due to increased confidence in their orthology resulting from comparisons of their exons and the relatively low fraction of sequence likely to be subjected to the scrutiny of natural selection [27]. Introns were sampled from all human autosomes so as to capture the genomic diversity of mutation processes. Sequence regions likely to evolve by a non-point mutation process, including low-complexity sequence and indels, were excluded in a manner that preserved the integrity of naturally occurring dinucleotides. The intron sequence alignments from the resulting sampled genes were broken into exactly 50 kbp long blocks to facilitate comparisons of parameters estimated from different alignments. Each alignment block was derived from a single gene but multiple alignment blocks may derive from the same gene. There were a total of 470 such alignments. (The full sampling protocol is presented in Methods.)

We contrasted comparable variants of the TF and NF forms for analysis of the real biological sequences by comparison to the corresponding nuGTR baseline parameterization. Given their relationships to an independent nucleotide process, the NF_nuGTR _baseline *is *a nucleotide GTR model while the TF _nuGTR _baseline *is not*. The only difference between the models being their differing weighting of elements of the instantaneous rate matrix by monomer and tuple frequencies respectively. The richest rate matrix parameterization considered was the diGTR, a fully general time reversible dinucleotide model. The diGTR model includes a parameter for each of the 48 instantaneous dinucleotide exchanges (conventionally one is omitted to calibrate the model, resulting in 47 free parameters). Because of considerable variation in both composition and substitution process across the genome of primates, we fit each model to each alignment independently and determined the support for the model across the entire data set as a cumulative ln *L *obtained as the sum of corresponding ln *L *from all alignments. See Methods for the complete model definitions, model implementation in software and procedure used for parameter estimation.

**Figure 2:**
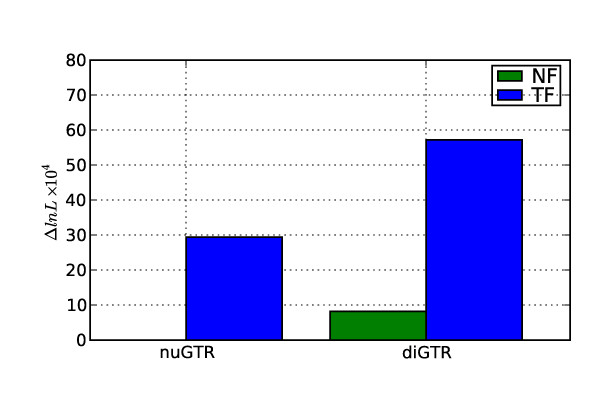
**Contrasting improvement of the dinucleotide GTR model over a nucleotide GTR process**. nuGTR – for NF this *is *the nucleotide GTR model, for TF it is a comparable parameterization; diGTR – fully general time reversible dinucleotide model, with 48 parameters in the rate matrix; Δ ln *L *– is the difference in the cumulative ln *L *from the alternate model compared to the cumulative ln *L *for the nuGTR model.

The magnitude of improvement over the nucleotide GTR model differed substantially between the TF and NF forms. Even the TF_nuGTR _model was 'better' than the fully parameterized NF _diGTR _model (Figure 2), despite the fact that for some alignments TF_nuGTR _was worse than the nucleotide GTR (results not shown). This property originates from the intrinsically context dependent nature of TF. The likelihood for the NF_diGTR _model still confers, however, a massive improvement over an independent nucleotide process, supporting the importance of a sequence neighborhood effect.

### Parameter estimates differ substantially between NF and TF forms

The MLE of a model parameter has particular significance since its relative position to the value 1 influences the interpretation concerning enhanced or suppressive influence of a context on substitution rate. We first examined the consistency in improvement over the nuGTR likelihood conferred by individual dinucleotide parameters. Each of the 48 distinct reversible dinucleotide exchanges was added to nuGTR for the TF and NF forms (see Methods). For example, we measured the influence of CpG to TpG substitutions, represented by the parameter *CG *⇔ *TG*, in models that also included the nucleotide GTR exchangeability parameters, denoting the resulting models NF_nuGTR+*CG*⇔*TG *_and TF_nuGTR+*CG*⇔*TG*_. The improvement conferred by the *CG *⇔ *TG *parameter was determined by contrasting the likelihood against that from the corresponding nuGTR model: NF_nuGTR _*vs *NF_nuGTR+*CG*⇔*TG*_; and, TF_nuGTR _*vs *TF_nuGTR+*CG*⇔*TG*_. As these comparisons are between nested models, the differences were measured using the standard likelihood ratio test statistic LRT. To facilitate comparisons between the two model forms, we further expressed the relative improvement conferred by each individual term as the proportion of that obtained for the diGTR. For example, the relative improvement over a nuGTR model conferred by the *CG *⇔ *TG *term is expressed as LR(*CG *⇔ *TG*)/LR(diGTR), where the ratio is between models belonging to the same model form.

**Figure 3:**
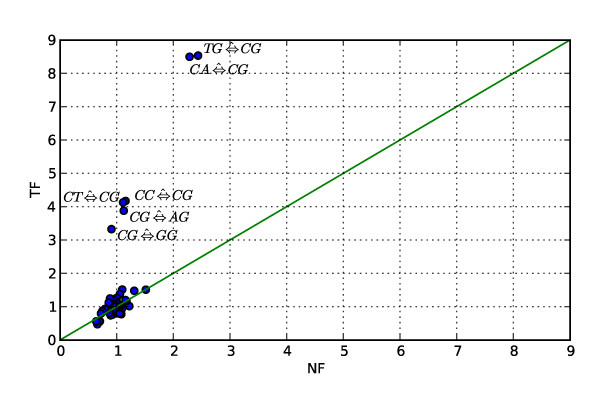
**Relationship between dinucleotide MLEs obtained under TF and NF from primate data**. Plotted are the mean MLEs for rate parameters obtained under NF_nuGTR+Parameter _(x-axis) and TF_nuGTR+Parameter _(y-axis) forms. Means and their standard errors were estimated using the jackknife procedure. Standard errors are not shown as they were smaller than the plotted marker sizes. Coordinates corresponding to the CpG related rate parameters are labelled.

**Figure 4:**
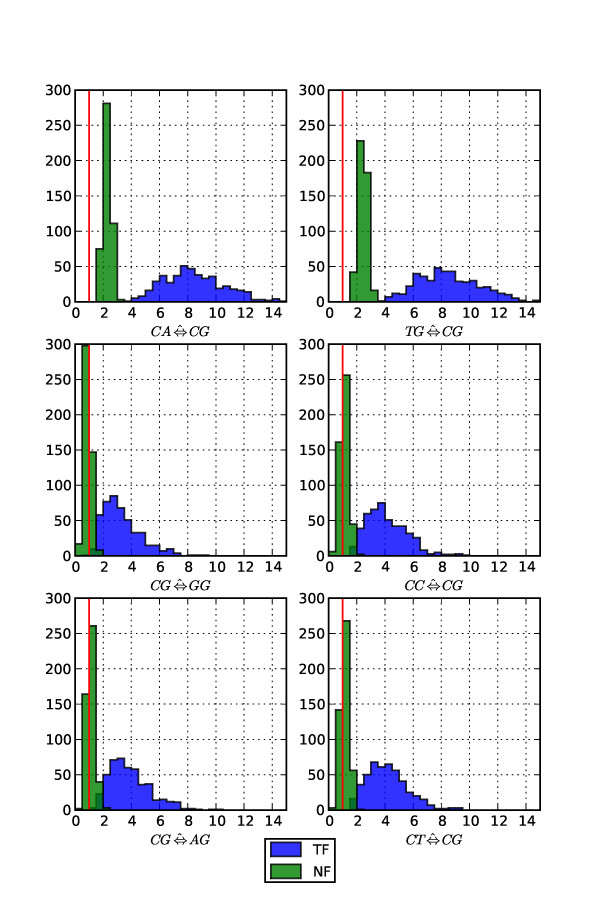
**CpG substitution rate MLEs were discordant between TF and NF**. The MLEs were those used to calculate the means in Figure 3. The vertical red line corresponds to the expected value under the null hypothesis (the parameter has no effect).

The contribution of each dinucleotide pair in improving model fit over nuGTR was assessed under both NF and TF forms. Dinucleotide parameters are listed in Table 1 by order of influence in an NF model. Whilst the seven most influential parameters were consistent between the NF and TF model forms, the order of importance was not preserved. The relative importance of dinucleotide parameters was reasonably consistent between the two forms, with CpG transition substitutions accounting for ~50% of the diGTR improvement over nuGTR. The degree of concordance between the mean MLEs obtained for the 48 parameters was considerable, but with striking outliers corresponding to the CpG terms (Figure 3). Of particular note is the observation that MLEs for CpG transversions were strongly discordant between the NF and TF forms. Under TF, CpG transversion MLEs are predominantly > 1, indicative of a greater rate of substitution than the background substitution rate (Figure 4). In contrast, under NF, the distribution of MLEs spans 1 (Figure 4) and the relatively small mean relative improvement percentage (≤ 0.5) along with a mean (per alignment) LR < 2 (Table 1) suggests CpG transversion rates are in fact not elevated at all, contradicting previous reports [13,16].

**Table 1:** Relative importance of dinucleotide parameters in improving the fit of the NF_nuGTR _or TF_nuGTR _model.

	**NF**			**TF**		
		
**Param**	$\bar{LR}$	$\bar{\%}$ (**SE**)	$\bar{Rank}$ (**SE**)	$\bar{LR}$	$\bar{\%}$ (**SE**)	$\bar{Rank}$ (**SE**)
*TG *⇔ *CG*	90.3	25.1 (0.3)	1.6 (0.0)	341.1	28.8 (0.2)	1.6 (0.0)
*CA *⇔ *CG*	85.8	23.7 (0.3)	1.7 (0.0)	366.7	30.9 (0.2)	1.4 (0.0)
*AT *⇔ *GT*	29.2	9.0 (0.2)	4.7 (0.1)	28.2	2.4 (0.1)	9.8 (0.2)
*AA *⇔ *GA*	25.8	7.9 (0.2)	5.0 (0.1)	47.5	4.1 (0.1)	6.9 (0.2)
*TT *⇔ *CT*	23.5	6.7 (0.1)	5.6 (0.1)	80.2	6.8 (0.1)	3.8 (0.1)
*AA *⇔ *AG*	19.4	5.5 (0.1)	6.7 (0.1)	55.6	4.7 (0.1)	5.5 (0.1)
*TT *⇔ *TC*	15.6	4.8 (0.1)	7.4 (0.1)	44.6	3.8 (0.1)	7.0 (0.2)
*GA *⇔ *GG*	14.3	3.8 (0.1)	9.4 (0.3)	16.5	1.3 (0.1)	20.9 (0.6)
*TC *⇔ *CC*	10.8	2.9 (0.1)	12.4 (0.4)	11.3	0.9 (0.1)	23.8 (0.6)
*AT *⇔ *AC*	10.1	3.2 (0.1)	11.2 (0.3)	19.0	1.6 (0.0)	12.8 (0.2)
*AG *⇔ *GG*	3.8	1.1 (0.1)	21.3 (0.5)	12.1	1.0 (0.1)	23.3 (0.6)
*CT *⇔ *GT*	2.8	0.8 (0.0)	24.2 (0.5)	1.5	0.1 (0.0)	35.1 (0.4)
*GC *⇔ *GA*	2.3	0.7 (0.0)	25.4 (0.5)	1.8	0.1 (0.0)	34.2 (0.4)
*AC *⇔ *GC*	2.1	0.6 (0.0)	26.6 (0.5)	17.8	1.5 (0.0)	13.8 (0.3)
*GC *⇔ *GG*	2.0	0.6 (0.0)	26.5 (0.5)	2.5	0.2 (0.0)	31.7 (0.4)
*CC *⇔ *CA*	1.9	0.6 (0.0)	27.2 (0.5)	2.6	0.2 (0.0)	30.9 (0.5)
*CT *⇔ *CA*	1.7	0.5 (0.0)	27.9 (0.5)	2.6	0.2 (0.0)	30.8 (0.5)
*AT *⇔ *AG*	1.7	0.5 (0.0)	27.8 (0.5)	2.8	0.2 (0.0)	29.5 (0.4)
*CT *⇔ *CG*	1.7	0.5 (0.0)	28.3 (0.5)	27.4	2.3 (0.1)	10.0 (0.2)
*CA *⇔ *GA*	1.7	0.5 (0.0)	28.5 (0.5)	4.8	0.4 (0.0)	25.1 (0.4)
*GT *⇔ *GA*	1.6	0.5 (0.0)	28.6 (0.5)	1.5	0.1 (0.0)	34.6 (0.4)
*CC *⇔ *CG*	1.5	0.4 (0.0)	29.8 (0.5)	26.1	2.2 (0.1)	10.0 (0.2)
*TT *⇔ *GT*	1.5	0.4 (0.0)	29.1 (0.5)	1.7	0.1 (0.0)	34.1 (0.4)
*TA *⇔ *TG*	1.5	0.5 (0.0)	29.4 (0.5)	3.1	0.3 (0.0)	30.8 (0.5)
*CT *⇔ *CC*	1.4	0.4 (0.0)	29.4 (0.5)	6.4	0.5 (0.0)	25.6 (0.5)
*CG *⇔ *AG*	1.4	0.4 (0.0)	29.6 (0.5)	23.7	2.0 (0.1)	11.1 (0.2)
*TC *⇔ *GC*	1.4	0.4 (0.0)	29.6 (0.5)	1.8	0.2 (0.0)	33.0 (0.4)
*GT *⇔ *GC*	1.4	0.4 (0.0)	29.2 (0.5)	10.4	0.9 (0.0)	19.1 (0.4)
*TT *⇔ *TG*	1.4	0.4 (0.0)	29.4 (0.5)	4.9	0.4 (0.0)	24.9 (0.4)
*GT *⇔ *GG*	1.3	0.4 (0.0)	29.9 (0.5)	2.4	0.2 (0.0)	31.5 (0.4)
*CG *⇔ *GG*	1.3	0.4 (0.0)	30.2 (0.5)	16.3	1.4 (0.0)	13.9 (0.3)
*TC *⇔ *AC*	1.3	0.4 (0.0)	29.6 (0.5)	1.7	0.2 (0.0)	33.5 (0.4)
*TA *⇔ *CA*	1.3	0.4 (0.0)	30.6 (0.5)	4.9	0.4 (0.0)	27.7 (0.5)
*AC *⇔ *AG*	1.3	0.4 (0.0)	30.6 (0.5)	1.3	0.1 (0.0)	35.5 (0.4)
*TC *⇔ *TG*	1.3	0.4 (0.0)	30.2 (0.5)	3.7	0.3 (0.0)	27.7 (0.5)
*TA *⇔ *AA*	1.2	0.4 (0.0)	30.7 (0.5)	1.6	0.1 (0.0)	34.4 (0.4)
*AT *⇔ *AA*	1.2	0.4 (0.0)	30.3 (0.5)	1.8	0.2 (0.0)	32.9 (0.4)
*TT *⇔ *AT*	1.2	0.4 (0.0)	30.6 (0.5)	1.8	0.2 (0.0)	33.0 (0.4)
*TG *⇔ *GG*	1.2	0.4 (0.0)	30.5 (0.5)	2.5	0.2 (0.0)	31.6 (0.4)
*TC *⇔ *TA*	1.2	0.4 (0.0)	31.0 (0.5)	1.5	0.1 (0.0)	35.1 (0.4)
*CT *⇔ *AT*	1.2	0.4 (0.0)	30.4 (0.5)	4.1	0.3 (0.0)	26.6 (0.5)
*CC *⇔ *GC*	1.2	0.4 (0.0)	30.9 (0.5)	1.7	0.1 (0.0)	34.2 (0.4)
*TT *⇔ *TA*	1.2	0.4 (0.0)	31.2 (0.5)	1.5	0.1 (0.0)	34.6 (0.4)
*TA *⇔ *GA*	1.2	0.4 (0.0)	31.1 (0.5)	1.6	0.1 (0.0)	34.9 (0.4)
*AC *⇔ *AA*	1.1	0.3 (0.0)	31.0 (0.5)	1.5	0.1 (0.0)	34.0 (0.4)
*CC *⇔ *AC*	1.1	0.4 (0.0)	31.2 (0.5)	2.5	0.2 (0.0)	31.3 (0.5)
*TG *⇔ *AG*	1.1	0.4 (0.0)	31.6 (0.5)	1.9	0.2 (0.0)	33.0 (0.4)
*CA *⇔ *AA*	1.1	0.3 (0.0)	30.9 (0.5)	2.9	0.2 (0.0)	29.7 (0.5)

The alternate dinucleotide models included the null model's nucleotide GTR terms plus the indicated dinucleotide parameter (Param). $\bar{\text{LR}}$ – the per alignment mean likelihood ratio of a nuGTR + Param against the corresponding nuGTR; $\bar{\%}$ (SE) – the per alignment mean (and standard error) relative importance of a parameter, expressed as a percentage of the diGTR LR; $\bar{\text{Rank}}$ (SE) – the per alignment mean (and standard error) rank of the LR. Means and standard errors were estimated using the jackknife procedure. The number of alignments was 470.

**Figure 5:**
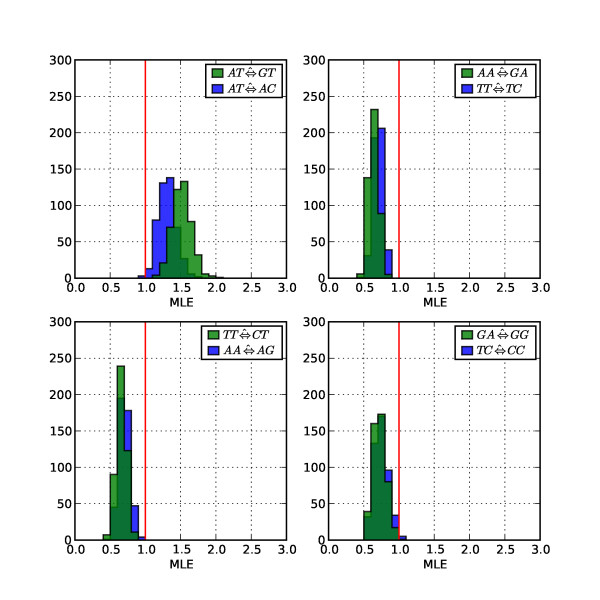
**Distribution of MLEs for non-CpG top 10 ranked dinucleotide parameters under NF**. x-axis – MLEs, these were the same as those used to calculate the means in Figure 3; y-axis – the number of alignments. The vertical red line corresponds to the expected value under the null hypothesis (the parameter has no effect). Strand complementary substitutions are plotted on the same chart.

More generally, the results from the NF model indicate that (after normalization) the 10 top-ranked parameters account for ~85% of the improvement conferred by the diGTR models 48 free parameters. Each parameter in the top 10 list was a transition substitution whose strand-complement was also in the top 10 list. Indicating that substitution processes were strand-asymmetric, the strand-complementary parameters were not always immediately adjacent in rank, e.g. *AT *⇔ *GT *was 3rd, while *AT *⇔ *AC *was 10th (Table 1, Figure 5).

## Discussion

The different rate matrix weighting of the TF and NF model forms have considerable influence on their statistical properties. Relative to an independent nucleotide process, when nucleotide frequencies are asymmetric, all exchangeability parameters under TF are confounded with sequence composition parameters. Our analysis of real biological data indicate that this difference is affecting inference. Although the relative importance of exchangeability parameters determined under either model form were related, striking discrepancies between the models were evident for important processes, indicating the parameters estimated under TF can be strongly misleading.

A key property of TF, that the stationary motif distribution readily becomes the observed distribution, is more difficult to achieve with NF. In real biological sequences, the frequencies of dinucleotide and higher order tuples are not products of their monomer frequencies. Accounting for this feature is a natural motivator in the TF model design where, by definition, the evolutionary process arrives at the observed distribution of sequence states. In contrast, specifying an NF model where the tuple frequencies are not just the product of monomer frequencies is more complex. One way it could be achieved is by specifying a non-reversible process, although the suitability of such a model for the commonly employed eigendecomposition matrix exponentiation algorithm remains to be established. Across a range of genomes, codon frequencies are reasonably approximated by position-specific nucleotide frequencies [28]. This suggests using position-specific nucleotide frequencies as a reasonable alternate approach, at least for protein coding sequences.

That the likelihoods achieved under TF are enormously improved compared to NF (Figure 2) provides a cautionary note against focussing solely on the likelihood as the basis for contrasting models. In the current case, a naive interpretation of the different likelihood magnitudes would lead to the conclusion that the TF forms were superior. The same conclusions are reached even when an information theoretic transformation, such as AIC, that takes account of the larger number of free parameters in TF is used (result not shown). That this apparent superiority over a simple model does not hold universally (e.g. ln *L *from TF_nuGTR _can be worse than that from nucleotide GTR) illustrates the challenge in using TF. It also highlights the necessity that consideration be given to whether the parameters estimated under a model make sense. Using the latter benchmark, however, requires knowing *a priori *the expected values of the parameters in the model.

One benchmark for establishing the suitability of a model form is how the desired null hypothesis is nested within it. We suggest that as context dependent models are intended to measure departures from independence, they should conveniently include an independent monomer process. As we have shown, for the TF form this is far from convenient. Comparable rate parameters between the TF and NF forms are only identical when the tuple frequencies are identical (see equation 3). For homogenous multiplicative *π *[*π *(*a*) = *π *(*i*_1_) *π *(*i*_2_)], at least 4 more exchangeability parameters are required for the TF variant to include the nucleotide GTR. From this it follows that simpler TF models, such as TF_nuGTR_, do not always contain the independent process but instead can be nested within the TF variant that does contain the independent process, substantially complicating inference of LRTs and the interpretability of exchangeability parameter MLEs. Contrast this with the NF form where NF_nuGTR _*is *the nucleotide GTR, NF_diGTR _is always measuring departures from independence and the context rate parameters (e.g. *CG *⇔ *TG*) in all NF models have, under the null of no effect, the expected value of 1 regardless of sequence composition. On this basis we suggest the NF form as a superior framework for the examination of context dependent effects.

Our analyses of real, substantively neutrally evolving biological sequences suggests that the use of nested models for measuring support ensures a modest consistency between the models forms. The ranking of likelihood ratios for comparable models were largely consistent between the two model forms (Table 1). However, the MLEs of some equivalently specified exchangeability parameters are worryingly inconsistent. This discordance was most apparent for CpG transversions (Figures 3 and 4), whose substitution rate was consistently greater than 1 for the TF model form, an observation previously reported from application of TF based models [13,16] which has been interpreted as indicating CpG transversions occur at a rate greater than background. This effect indicates that under TF, CpG transversion parameters are compensating for the deficit in CpG dinucleotides relative to their expected frequency. In contrast, under NF the MLE distributions (Figure 4) and relative importance (≤ 0.5%, Table 1) of CpG transversions indicate they are occurring in a context-independent manner. This fits with current biochemical knowledge which indicates deamination of 5 mC converts a 5 mC·G base pair into a T-G mismatch that, contingent on a repair failure, results in a C⇒T transition mutation.

The analysis of context dependent effects supports a strong influence of sequence neighborhood on mutation, evidenced by the significance of NF_diGTR _(Figure 2). Our assessment of the contributions to this fit from individual neighborhood under NF distinguishes nucleotide from neighborhood influences. Even after removing the hypermutable CpG effects, the NF model indicates that sequence neighborhoods are important. When we normalized the relative importance statistic (% column, Table 1) so they summed to 100, the top 10 ranked parameters account for ~85% of the fit achieved by the NF diGTR model, with CpG transitions alone accounting for ~46%. The etiology for these neighborhood influences is known for CpG transitions [2]. Six of the remaining eight parameters involve transition substitutions affecting the dipyrimidines CpC, CpT, TpC and TpT (and their strand complements). A candidate for the dipyrimidine effects is the dedicated repair system for repair of lesions arising at dipyrimidines [29,30] consistent with the slower than background rates of substitution (i.e. MLEs < 1, Figure 5) at these contexts. The strand asymmetry in support and MLEs of context effects is consistent with previous reports [31] and likely reflects the influence of transcription coupled DNA repair (TCR) processes, which selectively repair the transcribed strand [32]. Thus, although a specific candidate process for the remaining neighborhood influence (*AT *⇔ *GT*/*AT *⇔ *AC*) is not clear, the strong strand asymmetry of the MLEs indicate it is strongly affected by TCR and thus a target of either the base excision or nucleotide excision repair systems.

The substantial number of published independent tuple models which adopt the TF equilibrium distribution will display similar properties to those demonstrated here. Noteworthy, but by no means the only, examples of software implementing TF based models include the codon models of PAML [33], the doublet and codon models of Mr Bayes [14] and the dinucleotide and codon implementations in PyEvolve [23] and PyCogent versions up to 1.2 [34]. Software implementing the NF model forms include HyPhy [35] and PyCogent versions after 1.2.

## Conclusion

We have shown that models with NF form measure the effect of sequence neighborhood as departure from an independent nucleotide process, and that estimates of parameters within this model are robust to changes in sequence composition. In contrast, measurement of context dependent substitution influences with parameters from models with the TF form are confounded with sequence composition. We suggest, therefore, that results from models with the latter form be re-evaluated with a comparable NF model. Our application of NF dinucleotide models to primate introns confirms that sequence neighborhoods exert a strong influence on the rates of substitution and that transitions affecting CpG and dipyrimidine dinucleotides account for ~75% of the effect of immediate neighbors measured by the diGTR model.

## Methods

### Data

Aligned introns from the human, chimpanzee and macaque genomes were obtained from Ensembl release 49 [36]. Using coordinates of human genes we sampled a maximum of 100 alignments from each of 5 blocks of human autosomes (1–3, 4–6, 7–10, 11–15, and 16–22) to ensure representation of substitution processes across the primate genomes. As the substitution models represent the dynamics of point mutations only, any sequence that may have evolved by a non-point mutation process was replaced by an equivalent length run of '?'. Given their susceptibility to mutation by slipped strand mispairing, we specifically masked simple tandem-repeat sequences, including di- to tetra-nucleotide repeats with 5 or more repeat units. Mono-nucleotide repeats ≥ 10 nucleotides long were also masked. Sequence masking was done on the raw sequences (without gaps) while the locations of gaps were retained. Gaps were also masked as they arise by a non-point mutation process. The resulting masked alignments were then split into columns of non-overlapping dinucleotides which were filtered such that any column containing a dinucleotide made up of one or more of the characters 'N,?,-' was eliminated and the remaining columns were then joined to form the filtered alignments. Only alignments ≥ 50 kbp long were retained and these alignments were sliced into exactly 50 kbp long aligned blocks with any remaining sequence discarded. There were 470 such alignments.

### Model definitions

The following conditions were applied to all model definitions: all exchangeability matrices (*r*) were symmetric such that *r*(*a*, *b*) = *r*(*b*, *a*); diagonal elements of instantaneous rate matrices are determined by the constraint that the row sums are 0; and, the instantaneous rate matrices were scaled such that their trace = -1.

The nucleotide substitution models employed constitute variants of the rate matrix defined in the Results. Consider the nucleotide substitution rate matrix *q*, which has elements *q*(*i*, *j*) = *r*(*i*, *j*) *π *(*j*), where *i*, *j *∈ {A, C, G, T}. When *r*(*i*, *j*) = 1, ∀ *i *≠ *j*, we have the F81 substitution model [37], otherwise when *r*(*i*, *j*) ≠ 1, we have the general time reversible nucleotide substitution model [25,26]. For dinucleotides *a*, *b *made up of nucleotides *i*_1_*i*_2 _and *j*_1_*j*_2 _respectively, the NF_nuGTR _model rate matrix *Q*_nuGTR _has elements *Q*_nuGTR_(*a*, *b*) = *r*(*i*_1_, *j*_1_) *π *(*j*_1_) when dinucleotides *a*, *b *differ at the first position, *Q*_nuGTR_(*a*, *b*) = *r*(*i*_2_, *j*_2_) *π *(*j*_2_) when dinucleotides *a*, *b *differ at the second position, and *Q*_nuGTR_(*a*, *b*) = 0 when dinucleotides *a*, *b *differ at both positions. We illustrate the nuGTR + Param substitution models for the case of CpG/TpG exchanges. Under a nuGTR + *CG *⇔ *TG *model, *Q*_nuGTR+*CG*⇔*TG*_(*a*, *b*) = *r*(*CG*, *TG*)*Q*_nuGTR_(*a*, *b*) when {*a*, *b*} is {*CG*, *TG*} and *Q*_nuGTR+*CG*⇔*TG *_(*a*, *b*) = *Q*_nuGTR_(*a*, *b*) otherwise. The NF_diGTR _model rate matrix *Q*_diGTR _has elements *Q*_diGTR_(*a*, *b*) = *r*_diGTR_(*a*, *b*) *π *(*j*_1_) when dinucleotides *a*, *b *differ at the first position and *Q*_diGTR_(*a*, *b*) = *r*_diGTR_(*a*, *b*) *π *(*j*_2_) when they differ at the second position. The TF form dinucleotide models were defined similarly to the comparable NF ones, but replacing the *π *(*j*_1/2_) with *π *(*b*).

### Validation of Model Implementation

The accuracy of all software implementations of the different substitution model forms were subject to stringent validation. Three of the authors (HL, VBY and GAH) re-implemented both the TF and NF model classes substantively independently from the core PyCogent classes; only PyCogent's matrix exponentiation routines were common to the implementations. The correctness of this code was established by comparison against hand-calculated examples for species triplets. We relied on the theoretical relation between the independent nucleotide process and NF in the development of these calculations. In particular, when the null hypothesis of independence between nucleotides is true, that the matrix of dinucleotide substitution probabilities for time *t *is equal to the Kronecker product of the nested nucleotide substitution matrix for time *t*, i.e.. P_dinuc _= P_nuc _⊗ P_nuc_. We also relied on the fact that a TF model is equal to a NF model when the motif states are equi-frequent, and different otherwise. The results from a modest number of examples were also compared to those computed using R [38]. We then modified the existing PyCogent substitution model classes to allow specification of instantaneous rate matrices with the NF model form – where the *π *elements included in the rate matrix correspond to the probability of the ending nucleotide. The different implementations and indicated tests were used to validate the accuracy of the implementation in the modified PyCogent library.

### Simulations

We used a genomic alignment, processed as described above, of introns from the arbitrarily selected human gene ENSG00000003147 to estimate branch lengths on the unrooted tree relating human, chimpanzee and macaque. For Figure 1 a nucleotide GTR substitution model [25,26] was fitted and the resulting parameter MLEs used for simulation of 10 kbp alignments. The parameter MLEs were: '(human:0.014, chimpanzee:0.014, macaque:0.109)', (branch lengths rounded to the 4th decimal); nucleotide probabilities {*π *(*A*) ≈ 0.28, *π *(*T*) ≈ 0.32, *π *(*C*) ≈ 0.19, *π *(*G*) ≈ 0.21} and exchangeability parameters {*r*_*C*⇔*A *_≈ 0.20, *r*_*C*⇔*G *_≈ 0.34, *r*_*T*⇔*A *_≈ 0.12, *r*_*T*⇔*C *_≈ 0.71, *r*_*T*⇔*G *_≈ 0.19 and *r*_*A*⇔*G *_= 1}.

Simulations under the TF form were also done using parameters obtained from fitting ENSG00000003147. Specifically, the TF_nuGTR _model was fitted to the alignment and the resulting parameter MLEs used to simulate 100, 50 kbp long alignments. An important difference to the simulations was that dinucleotide (not nucleotide) frequencies were estimated from the alignment and used for simulation. All simulations were performed using PyCogent [34].

### Statistical testing

The substitution model classes of PyCogent [34] were modified to allow specification of these NF model form and this version of PyCogent is included as Additional data file 1. Maximum likelihood estimates of parameters were obtained for each alignment separately by numerical optimization using the built-in Powell optimization routine [39]. A tolerance of 10^-6 ^was applied with a maximum of 5 restarts. All LRT tests were executed using PyCogent.

### Availability of algorithms and data

The modifications to PyCogent implementing the NF model form will be available (on acceptance) as part of the standard open source PyCogent distribution [34]. They will appear in PyCogent versions greater than 1.2. (A modification of PyCogent version 1.1 with these new capabilities is included as Additional data file 1.) Details of the scripts used to simulate alignments and for model fitting are included in Additional data file 2. The simulated alignments and the masked and filtered primate intron alignments are available on request from the authors.

## Abbreviations

5 mC: 5-methyl-cytosine; diGTR: the most parameter rich dinucleotide substitution model; LR and LRT: likelihood ratio and likelihood ratio test; MLE: maximum likelihood estimate; NF: nucleotide frequency weighted rate matrix; nuGTR: the baseline dinucleotide substitution model, which included the nucleotide GTR terms; TCR: transcription coupled repair; TF: tuple frequency weighted rate matrix.

## Competing interests

The authors declare that they have no competing interests.

## Authors' contributions

HL contributed to study design, proofs, analysis and method implementation; VBY, contributed to study design, proofs, analysis and method implementation; HY contributed source code used for sampling mammal alignments; GAH conceived project, contributed to design, proofs, implementation, analyses and interpretation.

## Reviewers' comments

### Reviewer's report 1

Rob Knight, University of Colorado, Boulder

Models of molecular evolution are critical for providing the background against which adaptive evolution can be detected, and for understanding how mutational and selectional processes differ in different genomes (or parts of the same genome). In this manuscript, Lindsay et al. compare two widely used methods of incorporating context effects (i.e. the effects of neighboring nucleotides) in the standard Markovian model of molecular evolution: weighting by nucleotide frequencies (NF) and by tuple frequencies (TF). TF is very widely used in software such as the popular PAML and MrBayes phylogenetics packages. They are able to show both analytically and through simulations that the TF form introduces subtle biases due to sequence composition that could lead to incorrect biological conclusions: for example, the effect of methylation at CpG islands and subsequent deamination of the C to T is thought to be a key mutational pressure in mammals, but the TF form "identifies" this effect even under simulation conditions where it cannot exist. The study thus has important implications for other studies of molecular evolution (in particular, recommendation of NF rather than TF models), and may result in widespread evaluations of the degree of adaptation and the key evolutionary parameters in genes and genomes throughout the tree of life.

One interesting feature of this work is that the key parameters (as measured by order of importance) important for nucleotide substitution in mammalian introns differ substantially between the NF and TF forms on the same data. For example, the AT ↔ GT rate is third in importance with the NF model but seventh in importance with the TF model; the CG ↔ AG rate is tenth in importance with the TF model but 26th in importance with NF. These sorts of differences would lead to very different understandings of the relative contributions of different kinds of mutations and inferences about the molecular mechanisms involved. One addition that would make this table easier to interpret would be the addition of errors on the % importance and rank importance obtained by bootstrapping the data set if this would take an acceptable amount of computation time – for example, how large a change in rank is meaningful?

***Author's response***: *We thank the reviewer for this suggestion and have modified the Table to include estimates of the variability in both relative importance (%) and rank. These were obtained using a jackknife resampling procedure. The associated text in the manuscript has also been revised*.

Two other additions that would be useful, but perhaps best left for future work, would be (i) more detail about the kinds of molecular mechanisms (deamination, G oxidation, repair pathways, etc.) that are most likely to be prone to over- or under-representation using the TF model, and (ii) extension of the work beyond mammals. Mutational processes are of intense medical interest in studies of retroviral adaptation, for example. I also note that my 2001 Genome Biology paper (PMID 11305938) indicates that the product of position-specific nucleotide frequencies does in fact recapture codon frequencies extremely well across a wide range of genomes, so the authors might want to use that argument to bolster the applicability of their modeling assumptions.

***Author's response***: *We can address the first point only in those cases where a strong candidate mechanism has been identified for a context. The dominant influence of deamination of 5-methyl-cytosine on genomic composition (depletion of CpG) appears responsible for the high ranking of CpG transversions under the TF form. Aside from this obvious example of cytosine deamination, the association between the context effects and their causative mutagenic processes is poor, limiting our ability to comment further*.

*We thank the reviewer for bringing to our attention this finding from his Genome Biology paper. Codon models are certainly the most popular application of context dependent models and the result concerning the consistency of observed codon frequencies with those calculated from position-specific nucleotide frequencies provides strong guidance for model design. We now include this reference in our discussion of the different properties of the NF/TF models*.

Overall, I believe that this paper is an important contribution and is likely to lead to much wider development and application of NF models in future.

### Reviewer's report 2

Joshua Cherry, NCBI (nominated by David Lipman)

This article makes an important point: some models of the TF form, including some widely used models, are inadequate for many purposes and can yield biased results. However, it unfairly and incorrectly impugns TF in several places. It seems to claim that TF, unlike NF, cannot represent independence of sites in the face of unequal base frequencies, but this is only true of special cases of TF. In fact the most general TF model contains every NF case as a submodel, so TF can model any case that NF can model (and more). Furthermore, a simplified example is incorrectly alleged to demonstrate bias of estimates based on TF. Also, NF has its own weaknesses, and the best model for a particular purpose might conform to neither NF nor TF. The sweeping conclusions that "models with the TF form are systematically biased by sequence composition" (Conclusions) and "the NF form should be used" (Abstract) are unjustified.

***Author's response***: *We agree with the reviewer that the TF form can also represent independence of sites when base frequencies are unequal and have now revised substantial sections of the results and discussion to better explain the relationship between these two model forms. We also agree that the NF form has its own weaknesses, and detail these in the discussion. We have also toned down statements concerning systematic bias. We dispute, however, that we are not justified in claiming the NF form should be used. We elaborate on each of these issues in our response to the reviewer's detailed comments below*.

Paragraph 3 of Results seems to argue that when base frequencies are unequal, TF cannot represent cases where changes are independent of sequence context. This is not true of all TF models. The argument that *π *(T) *π *(G) is in general different from *π *(C) *π *(G) ignores the *r*_TF _terms, which may be such that the rates are equal. The most general TF model contains the model with independence as a submodel, so it clearly can represent that case and would give consistent estimates of deviation from independence. In short, some TF models can model independence and are suitable for measuring context effects, even when there is compositional bias.

***Author's response***: *We agree that TF can represent cases where changes are independent of sequence context and there are unequal base frequencies. We have revised the manuscript to clarify this and the role of the r*_TF _*terms. Our point is, while *NF_nuGTR _*is the nucleotide GTR process*, TF_nuGTR _*is not, although one would expect it to be so. As a consequence, comparing *NF_nuGTR _*and *NF_diGTR _*is correct and clearly interpretable, at least for models with multiplicative π, and to a lesser extent for others, whereas TF is cumbersome: one needs to spend quite a lot of effort to specify the nucleotide GTR process, even after the unintuitive realization that *TF_nuGTR _*doesn't do the job*.

The above illustrates a more general point. A model need not be a case of TF in order to be nested in a case of TF. Every NF case is nested in the most general TF model, and may be nested in more restricted TF models as well. A model that only allows independence of sites may not be a TF model, but it is nested in a TF model. Since we are interested in models that allow context dependence, we are interested in more general models anyway.

***Author's response***: *We disagree with the last point. We are interested in context dependence in order to understand what contributes to it, not simply identifying its existence. We also suggest that models whose parameters do not affect the likelihood when the null of independence is true are superior to the alternative. This condition is satisfied by the NF form, not the TF form*.

Paragraphs 4–6 of Results claim to prove, using a simplified case with a two-letter alphabet, that TF leads to biased estimates when equilibrium frequencies are unequal. No bias is actually demonstrated, even for the special case of TF used. The intent in constructing the NF model seems to be that *κ *is a measure of a certain type of context effect, with *κ *= 1 corresponding to independence (zero context effect). For the TF model, the same *r *matrix is used. The values of *κ *estimated for the TF model are indeed quite different from 1 despite independence, but this is not an indication of bias. Rather, the *κ *in the TF model is a different parameter than the *κ *in the NF model, and is not expected to be 1 in this case. The *Q*_TF _matrix is capable of perfectly modeling the independent case. It does so with *κ *≠ 1 when frequencies are unequal. The estimated *κ *values are not biased, but are (in expectation, asymptotically) precisely what is required to yield a *Q*_TF _matrix that models the data. Modulo multiplication by a constant, the NF and TF models produce the same matrix values, but using different parameterizations.

***Author's response***: *We agree that the term 'bias' is not correctly applied here and have revised this portion of the manuscript. We also emphatically agree that κ in the two models is a different parameter when the sequence states **are **unequal, but exactly the same parameter when the sequence states are equal. This is the point of the section, and a major point of the manuscript. In most applications, it is the r terms from NF models that are being interpreted. Thus, even if an identical likelihood were to result from application of NF and TF models, the biological inference drawn from those models could be markedly different based on whether an r term was *< 1, = 1, > 1.

This would all be clearer if the parameter in the *Q*_TF _matrix were given a different name, e.g., *λ*. The estimates of *λ *do not cluster around 1, but these are not biased estimates of *κ*: they are asymptotically unbiased estimates of *λ*. To interpret *λ *≠ 1 as an indication of context effects is a human error. Whether there are context effects can be determined from consideration of the estimates of *λ *and *π *(R). This takes a bit more effort, but inconvenience is quite different from bias.

***Author's response***: *We have renamed the parameter to *K.

I am unsure why the *Q*_TF _matrix was given in terms of *π *(X) *π *(Y) rather than *π*_dinuc _(XY). The latter corresponds to eq. 2, and yields a different, more general model (modulo multiplication by a constant, it contains the *Q*_NF _matrix as a submodel).

***Author's response:****Our point was to demonstrate with a simple case the conditions where κ *= K* could occur. We have clarified this section of the manuscript*.

I am not sure that the other analyses are entirely fair to TF. The authors apparently design an NF model that is appropriate and then transfer its *r *values to TF to yield a model that is inappropriate. Perhaps it is equally possible to design an appropriate TF model, and transfer of its *r *values to NF would yield an inappropriate model (this must be true for some underlying true models). Along these lines, for the case of intron sequence evolution the authors consider the most general time reversible dinucleotide model. This model contains the others, and appears to justify its extra parameters with sufficient likelihood improvement. Why do the authors not simply take the results of this model as their best estimates and recommend the use of this TF model for this application?

***Author's response:****We accept the reviewer's suggestion of defining a TF model and simulating under this and then transferring the parameters to an NF model. This is now presented in the last paragraph of Results section on 'The relationship of NF and TF to an independent nucleotide process'*.

*Regarding our analysis of intron sequence evolution, we do not take the fully general model purely on the face value of its likelihood improvement because we are not interested in just identifying the best fitting model. Instead, we seek to understand the contributions of the different terms to the fit of this model, and the consistency between the models in interpreting their effects. We demonstrate there is broad consensus between the two forms, but that they differ with respect to key parameters that strongly influence our inference concerning the significance of a mutagenic process – namely the rate of transversions at CpG dinucleotides. We also point out that some potential applications of these models, such as phylogenetic reconstruction, would substantially benefit from using parameterizations that are less computationally intensive to fit than diGTR but succinctly capture the dominant effects*.

***Cherry responds in a second review***: This new paragraph, which mainly illustrates that a true null hypothesis is not often rejected, does not address the point that I was trying to make. In fact it is another example of the phenomenon that I was criticizing. Rather than using a properly designed TF model for assessing context effects, here and elsewhere the authors use an inappropriate TF model, TF_nuGTR_, obtained by blind transfer of the exchangeability matrix from a working NF model. They then blame TF in general for the result, and conclude, incorrectly, that TF cannot be used for the intended purpose.

It is possible, reasonable, and likely often desirable to use TF models to study context effects. The null hypothesis of independent nucleotide GTR can be expressed in TF form (as can anything that can be expressed in NF form, according to the definitions in the revised manuscript). This TF form is different from what the authors misleadingly call TF_nuGTR_. That may be surprising, but nonetheless it is possible to use a correct null hypothesis rather than TF_nuGTR_. This null hypothesis can be tested against the alternative hypothesis TF_diGTR_. This procedure will detect context effects when they are present but not when they are absent, subject to the usual statistical uncertainties.

It is unimportant whether the null hypothesis is expressed in TF form: the NF form or the single-nucleotide form will yield the same likelihood. The important point is that TF_diGTR _is a legitimate and possibly very useful non-null model. Indeed, as I noted above, it appears to be quite useful for modelling the intron sequence data analyzed in the article. Any extra effort required to interpret the parameters is the price that one pays for using a richer model that is capable of representing biological reality. Sometimes a less general model will be preferable, but the same can be said of any general model (e.g., single-nucleotide GTR). I see no valid argument for banishing this useful tool from our arsenal of models.

***Author's response:****Regarding the reviewer's statement concerning our specification of an "inappropriate TF model", we agree that is inappropriate but point out that this form was not used as a soft target. This parameterization was motivated by analogous TF form codon model parameterizations, specifically those of the Goldman and Yang substitution model family and subsequent refinements. Thus, the TF*_nuGTR_*model choice is pertinent to current applications*.

Regarding the reviewers suggestion that TF models are "reasonable, and likely often desirable" for the study of context effects, we illustrate again the additional complexity necessary to relate a TF model form to the relatively simple F81 substitution model. To parameterize F81 in TF, we need to set

$$r_{\text{TF}}(i_{1}i_{2},j_{1}j_{2})=\{\begin{array}{ll} 1/\pi (j_{1}) & i_{1}=j_{1}\\ 1/\pi (j_{2}) & i_{2}=j_{2} \end{array}$$

*thus apparently requiring 4 extra parameters, which are in fact functions of π, and the form of Q is harder to comprehend than the analogous expression for the NF model form, where there are no additional parameters and hence the r*_NF _*all equal 1*.

*Concerning the unimportance of the form of the null, we suggest it is enormously relevant to know which model actually corresponds to the desired null. We have argued that modelling context dependence can be done sensibly when an appropriate null can be identified, and that this null is obvious under the NF form. We claim for TF that even if a more restricted TF model than TF*_diGTR_*nests the independent process, identifying this model as the null for measuring context effects from the myriad possibilities is decidedly non-obvious*.

***Cherry's first review continues***: Paragraph 2 of the Discussion states that "...specifying an NF model where the tuple frequencies are not just the product of monomer frequencies is more complex. One way it could be achieved is by specifying a non-reversible process...." I would expand on this. I believe the following to be true, but not necessarily obvious:

1. If *r*_NF_(*a*, *b*) is symmetric, the equilibrium tuple frequencies equal the products of the nucleotide equilibrium frequencies, but

2. Symmetry of *r*_NF_(*a*, *b*) is not necessary (though it is sufficient) for time reversibility.

I would also note that NF, unlike TF, cannot represent different equilibrium frequencies at different tuple positions. This would seem to be a serious deficit for codon-based models. One can imagine an extension to NF in which there are distinct frequency parameters for each tuple position. I am unsure of the relationship of this extension to TF.

***Author's response:****We thank the reviewer for their interesting suggestion regarding asymmetric r. We have not modified the Discussion as we feel the existing text is adequate*.

*We agree that the NF form presented in the manuscript cannot represent position-specific equilibrium frequencies. However, as suggested by the reviewer, specifying independent and non-identical nucleotide frequencies is easy. Such models have already been described (e.g. Pond SK, Muse SV: Site-to-site variation of synonymous substitution rates. Mol Biol Evol 2005, 22:2375–2385). An argument for the suitability of this model form for the codon case was made by the first reviewer (R Knight, see above)*.

All that prevents any NF model from qualifying as a TF model is the restriction that *r*_TF _does not depend on *π*_dinuc_. Perhaps people tend to create this sort of model, which would make it worth analyzing. However, there is no reason for models to have this restriction. Even in single-base models, the analogous restriction is not always observed: in the F84 (Felsenstein, 1984) model, for example, some of the *r *values depend on *π *values. Perhaps the best models for some purposes are TF-like models that violate this restriction.

***Author's response:****We concede that there may be some purposes for which the TF-like models may be better suited. Our focus has been on establishing how to measure departures from independence, and in particular the identification of primary influences in departure from independence in the evolution of DNA sequences. A motivator for this is the intra-genomic diversity in composition evident for many organisms, including the primate species used for the intron analysis. In such a case, using a model in which the critical parameters are independent of composition seems the best choice. Under the NF form, parameters estimated from genomic regions that differ in composition can be readily compared. As pointed out by the reviewer, similarly defined parameters estimated under the TF form from the same regions can also be compared. However, the failure to appreciate the influence of composition on the latter estimates can strongly mislead their interpretation*.

### Reviewer's report 3

Stephen Altschul, NCBI (nominated by David Lipman)

This paper compares two different, widely used models used for studying the evolution of nucleic acids. A specific question is whether specific nucleotide substitutions depend upon the context in which they occur, and in what way. This is addressed by modeling the evolution of non-overlapping nucleotide "tuples", and comparing the results to a model of independent nucleotide evolution.

A structure imposed on such models assumes an instantaneous a→b tuple mutation rate expressible as a symmetric matrix on a and b, multiplied by a factor dependent upon the equilibrium frequency distribution of either the resulting tuple (b) or the resulting nucleotide in the mutation that occurs. The nucleotide-frequency normalized model is called NF, the tuple-frequency normalized model TF.

A central point of the paper is that for NF, a null model is exactly the same as a model of independent nucleotide evolution, whereas this is true for TF only when the equilibrium distributions of the nucleotides are identical. When there are non-equal nucleotide frequencies, TF can be seen to imply context-dependent factors in nucleic acid evolution when there are none, and sometimes to miss detecting such factors when they exist. The NF model does not have equivalent difficulties.

***Author's response:****We note here that the last point relates to an earlier version of the manuscript*.

Applied to real, non-protein-coding sequences, the NF and TF models both recognize very significant context-dependent effects on nucleic acid evolution, and agree on the most important dinucleotide pairs implicated. However, the models can diverge substantially concerning more subtle issues. Specifically, the NF model does not recognize a significant context-dependent influence on transversions involving CpG dinucleotides, whereas the TF model does.

In conclusion, this paper presents a provocative critique of the widely used TF model, and of some of the conclusions that have been derived therefrom. Its arguments will need to be taken into account in further modeling of nucleic acid evolution.

## Supplementary Material

Additional file 1 Modified version of PyCogent library. Archive of the PyCogent library developed for this study from PyCogent version 1.1. Click here for file

Additional file 2 Scripts used in the study. Archive of stand-alone web site presenting the central scripts used in this study. Click here for file
