# Pitfalls of the most commonly used models of context dependent substitution

**DOI:** 10.1186/1745-6150-4-10

**Published:** 2009-03-18

**Authors:** Helen Lindsay, Von Bing Yap, Hua Ying, Gavin A Huttley

**Affiliations:** 1 Computational Genomics Laboratory, John Curtin School of Medical Research, The Australian National University, Canberra, ACT 0200, Australia; 2 Department of Statistics and Applied Probability, National University of Singapore, Kent Ridge, Singapore

## Abstract

Correction to Lindsay H, Yap VB, Ying H, Huttley GA: Pitfalls of the most commonly used models of context dependent substitution. Biology Direct 2008, 3:52

## Correction

The published version of this article [1] includes a link to the incorrect version of 'Additional File Two'. The correct version of the file is included here as Additional file 1.

## Supplementary Material

Additional file 1 **Scripts used in the study.** Archive of stand-alone web site presenting the central scripts used in this study. Click here for file
