# Supplementary material for: Pitfalls of the most commonly used models of context dependent substitution
Source: Biol Direct. 2008 Dec 16;3:52. doi: 10.1186/1745-6150-3-52 (PMC2628887; doi:10.1186/1745-6150-3-52)
Supplement: Additional file 2 — Scripts used in the study. Archive of stand-alone web site presenting the central scripts used in this study. [file 1745-6150-3-52-S2.zip › HuttleyAdditional2/html/filter_masked_align.html]

Filtering masked alignments — Context Dependent Substitutions v-Draft documentation


### Navigation

- index
- next |
- previous |
- Context Dependent Substitutions v-Draft documentation »

# Filtering masked alignments¶

Sequence alignments were obtained from Ensembl release 49 that corresponded to the coordinates of a human gene for the three primate species of human, chimpanzee and macaque. For a sequence from a given species, any region corresponding to an exon was masked. We also masked low complexity sequence, and gaps. Masking was done on the original sequence with the placement of gaps preserved. Masked alignments were then filtered in dinucleotide frame so dinucleotide columns contained motifs consisting only of valid nucleotide characters. This sampling strategy ensured that only 16 dinucleotide states were present in the data.

The modules responsible for the filtering of masked alignment procedures are displayed below. The module for filtering the masked alignments (filter\_masked\_aligns.py) was:

```
import os, re, sys, subprocess
from optparse import OptionParser

expanduser = os.path.expanduser

from cogent import LoadSeqs, DNA
from cogent.parse.fasta import LabelParser
import mask_repeat

def repeat_mask_sample(input_dir, output_dir, b, e, aln_size, num_alns):
    """read in existing alignments and replace STR runs with the mask
    character, writing out the masked, filtered alignment"""
    
    if not os.path.exists(output_dir):
        r = subprocess.call(["mkdir", "-p", output_dir])
        if r != 0:
            print "Failed creating", output_dir
            sys.exit(-1)
    
    filenames = [fn for fn in os.listdir(input_dir) if fn.endswith(".fasta")]
    # we create a name parser for
    # >Homo sapiens:chromosome:7:8119341-8268710:-1
    latin = ['Homo sapiens', 'Pan troglodytes', 'Macaca mulatta']
    common = ['human', 'chimpanzee', 'macaque']
    
    common_name = lambda x: dict(zip(latin, common))[x]
    name_parser=LabelParser("%(Common)s",[(0,"Common",common_name)])
    c = 0
    for fn in filenames[b:e]:
        aln = LoadSeqs(os.path.join(input_dir, fn),
                        name_conversion_f=name_parser, moltype=DNA)
        aln = aln.takeSeqs(common)
        filtered_aln = mask_repeat.masked_filtered_aln(aln)
        for end in range(aln_size, len(filtered_aln), aln_size):
            begin = end - aln_size
            sub_aln = filtered_aln[begin:end]
            if len(sub_aln) < aln_size:
                continue
            
            name = "%s-%d-%d.fasta" % (fn.replace(".fasta",""), begin, end)
            aln_filename = os.path.join(output_dir, name)
            print "Wrote", aln_filename
            sub_aln.writeToFile(aln_filename)
            c += 1
            if c >= num_alns:
                exit("Num written = %s" % c)
            
        
    
    exit("Num written = %s" % c)

def exit(msg):
    print msg
    print "\nDone!"
    sys.exit(0)

if __name__ == "__main__":
    parser = OptionParser()
    parser.add_option("--input_dir", type="string",
                help="the input directory path with masked data")
    parser.add_option("--output_dir", type="string",
                help="the output directory path to write masked/filtered data")
    parser.add_option("--num_alns",
                help="the range of alignments to filter")
    parser.add_option("--aln_size", type="int",
                help="the size of the sought filtered alignment blocks")
    (options, args) = parser.parse_args()
    
    b, e = map(int, options.num_alns.split('-'))
    repeat_mask_sample(expanduser(options.input_dir),
                expanduser(options.output_dir), b, e, options.aln_size,
                options.num_alns)
    print "\nDone!"
```

The following module (mask\_repeat.py) was used for identifying repeated DNA sequences:

```
from __future__ import division
import os, re, sys
from optparse import OptionParser

from cogent import LoadSeqs, DNA

def make_words():
    """make the set of DNA words, up to k=4 and corresponding regexes to find
    runs of words"""
    def all_motifs(motif_length):
        vals = []
        for i in range(motif_length):
            current = []
            if not vals:
                vals = list('ACGT')
                continue
            for l in 'ACGT':
                for val in vals:
                    current.append("".join([val, l]))
            vals = current
        
        # we exclude mononucleotide runs, treating them separately
        keep = []
        for motif in vals:
            if len(set(motif)) > 1:
                keep.append(motif)
        return keep
    motifs = all_motifs(2) + all_motifs(3) + all_motifs(4)
    # turn into regexes
    template = "(%s){5,}"
    regexes = [re.compile(template % word) for word in motifs]
    keyed_regexes = zip(motifs, regexes)
    
    for nuc in "ACGT":
        keyed_regexes.append((nuc, re.compile("(%s){10,}" % nuc)))
    return keyed_regexes

all_regexes = make_words()

def replace_match(replace_with="?"):
    """callback function for the regex sub method. Determines the span of a
    match and returns a string of just N's of equal length."""
    def call(match):
        return replace_with * (match.end()-match.start())
    return call

def replace_repeats(data, replace_with="?"):
    """Replaces all di-tetra nucleotide repeats > 5 units long by ?'s"""
    replacer = replace_match(replace_with)
    for word, regex in all_regexes:
        data = regex.sub(replacer, data)
    return data

def repeat_masked_aln(aln, replace_with="?"):
    """for each seq in aln, substitute the STRs with replace_with char"""
    # this has the effect of masking STRs without affecting the placement of
    # gaps
    for seq in aln.Seqs:
        new = replace_repeats(seq.data._seq, replace_with=replace_with)
        seq.data._seq = new
    return aln

_valid_chars = lambda x: set(''.join(x)) <= set('ACGT')

def masked_filtered_aln(aln, replace_with="?"):
    """filter a masked alignment to exclude dinucleotides with non-nuc
    monomers, eg. exclude dinucleotide columns with 'A-' or '--', or
    'NN'."""
    aln = repeat_masked_aln(aln, replace_with=replace_with)
    motif_length=2
    assert motif_length == 2 # this is a dinucleotide project
    filtered_aln = aln.filtered(_valid_chars, motif_length=motif_length,
                                log_warnings=False)
    return filtered_aln

def test():
    """tests the masking funcs"""
    got = replace_repeats("CATACATACACACACACACACCCCACACATTTTT")
    assert got == "CATACATA????????????CCCCACACATTTTT", got
    got = replace_repeats("CATACATACACACCCCCCCCCCCCACACATTTTT")
    assert got == "CATACATACACA????????????ACACATTTTT", got
    
    data = [('a', "CATACATACACACACACACACCCCACACATTTTT"),
            ('b', "CATACATACACACACACACACCCCACACATTTTT")]
    aln = LoadSeqs(data=data)
    masked = repeat_masked_aln(aln)
    seqs = ' '.join(masked.todict().values())
    expect = " ".join(['CATACATA????????????CCCCACACATTTTT',
                       'CATACATA????????????CCCCACACATTTTT'])
    # we shouldn't have any repeat
    for repeat, regex in all_regexes:
        assert replace_repeats(seqs) == expect
    
    # filtering a masked alignment should return complete dinucleotides
    # that existed in the original sequence and exclude any that contain
    # the ? character
    filt_aln = masked_filtered_aln(aln)
    expect = ['CATACATACCCCACACATTTTT',
              'CATACATACCCCACACATTTTT']
    expect = zip('ab', expect)
    assert str(LoadSeqs(data=expect)) == str(filt_aln)
    print 'Tests passed\n'

def run(b, e, input_dir, output_dir, aln_size, motif_length):
    filenames = [f for f in os.listdir(input_dir) if f.endswith(".fasta")]
    num_written = 0
    mask_chars = lambda x: not set('-N?') & set(''.join(x))
    
    for fn in filenames[b:e]:
        print "Doing", fn
        aln = LoadSeqs(os.path.join(input_dir, fn), moltype=DNA)
        aln = repeat_masked_aln(aln)
        aln = aln.filtered(mask_chars, motif_length=motif_length)
        for end in range(aln_size, len(aln), aln_size):
            begin = end - aln_size
            sub_aln = aln[begin:end]
            if len(sub_aln) < aln_size:
                continue
            name = "%s-%d-%d.fasta" % (fn.replace(".fasta",""), begin, end)
            aln_filename = os.path.join(output_dir, name)
            sub_aln.writeToFile(aln_filename)
            num_written += 1
            print "Wrote #=%d; %s" % (num_written, aln_filename)
    

def fail(parser):
    """print help and exit"""
    print "Failed:"
    parser.print_help()
    sys.exit(-1)

if __name__ == "__main__":
    parser = OptionParser()
    parser.add_option("--test", action="store_true", default=False,
                help="runs test set")
    parser.add_option("-d", "--align_nums", dest="align_nums", default=None,
                type="string",
                help="the alignment number to compute, or a range (eg 0-10)")
    parser.add_option("--input_dir", type="string",
                default=None,
                help="path containing individual input alignment files")
    parser.add_option("--output_dir", type="string", default=None,
                help="path to write filtered alignment files")
    parser.add_option("--aln_size", type="int", default=None,
                help="size of alignments to write.")
    #
    parser.add_option("--motif_length", type="int", default=2,
                help="motif length, default=2")
    (options, args) = parser.parse_args()
    
    if options.test:
        test()
        exit()
    
    if options.align_nums is None:
        fail(parser)
    
    if '-' in options.align_nums:
        align_nums = map(int, options.align_nums.split('-'))
    else:
        align_nums = int(options.align_nums)
        align_nums = (align_nums, align_nums+1)
    
    run(align_nums[0], align_nums[1], options.input_dir, options.output_dir, options.aln_size, options.motif_length)
```

#### Previous topic

Parameters estimated from TF models are affected by composition

#### Next topic

Fitting the dinucleotide GTR model

### This Page

- Show Source

### Quick search

### Navigation

- index
- next |
- previous |
- Context Dependent Substitutions v-Draft documentation »

© Copyright 2008, Gavin Huttley.
Last updated on Dec 09, 2008.
Created using Sphinx 0.5.
