# Supplementary material for: Pitfalls of the most commonly used models of context dependent substitution
Source: Biol Direct. 2008 Dec 16;3:52. doi: 10.1186/1745-6150-3-52 (PMC2628887; doi:10.1186/1745-6150-3-52)
Supplement: Additional file 2 — Scripts used in the study. Archive of stand-alone web site presenting the central scripts used in this study. [file 1745-6150-3-52-S2.zip › HuttleyAdditional2/html/tf_lnL_worse.html]

The likelihood of the TF form can be worse than that of a comparble nucleotide model — Context Dependent Substitutions v-Draft documentation


### Navigation

- index
- modules |
- next |
- previous |
- Context Dependent Substitutions v-Draft documentation »

# The likelihood of the TF form can be worse than that of a comparble nucleotide model¶

Given the TF model form is not an independent nucleotide process, it is possible that the likelihood of a TF model on an alignment will be worse than a nucleotide model with similar exchangeability parameters.

We prove this here by example.

```
>>> from cogent import LoadSeqs, LoadTree, DNA, LoadTable
>>> from cogent.evolve.substitution_model import Nucleotide
```

We use a phylogenetic tree and nucleotide composition estimated from an alignment of human, chimpanzee and macaque from an alignment of orthologous introns from ENSG00000002822 (positions 0 - 500000).

```
>>> tree = LoadTree(treestring='(human:0.00986214818868,chimpanzee:0.00962734349165,macaque:0.0807583420651)')
>>> nuc_probs = {'A': 0.18595999999999999,
...              'C': 0.26468666666666668,
...              'T': 0.25644,
...              'G': 0.29291333333333336}
```

We define a F81 substitution model, and likelihood function using these values and store a reference to the simulateAlignment method, just to simplify the code. We will be simulating alignments of 10Kbp length.

```
>>> nt = Nucleotide(motif_probs=nuc_probs)
>>> lf = nt.makeLikelihoodFunction(tree)
>>> sim_align = lf.simulateAlignment
>>> sequence_length = 10000
```

We then define a F81 substitution model and likelihood function that will be used for estimation from the simulated alignments,

```
>>> nt = Nucleotide()
>>> nuc_lf = nt.makeLikelihoodFunction(tree)
```

and a TF based dinucleotide substitution model and likelihood function that will also be used for estimation from the simulated alignments. This TF model form weights exchanges by the frequency of the end state dinucleotide. Given the value of nuc\_probs (above), the dinucleotide frequencies will be unequal. The use\_monomer\_probs=False argument specifies this as a TF model form.

```
>>> tf_dinuc = Nucleotide(motif_length=2, use_monomer_probs=False)
>>> tf_lf = tf_dinuc.makeLikelihoodFunction(tree)
```

We simulate 1000 alignments. For each alignment we separately fit the F81 and TF models to the same alignment and take the likelihood ratio. The same optimisation settings were used for fitting both models: a global (simulated annealing) optimiser first followed by the local (Powell) optimiser. The ratio of the likelihoods estimated from each alignment was recorded.

```
>>> LRs = []
>>> for i in range(1000):
...     print i
...     aln = sim_align(sequence_length=sequence_length)
...     nuc_lf.setAlignment(aln)
...     nuc_lf.optimise(max_restarts=5, show_progress=False)
...     nuc_lnL = nuc_lf.getLogLikelihood()
...     tf_lf.setAlignment(aln)
...     tf_lf.optimise(max_restarts=5, show_progress=False)
...     dinuc_lnL = tf_lf.getLogLikelihood()
...     LRs += [[dinuc_lnL-nuc_lnL]]
```

We save the results to a file for display later.

```
>>> table = LoadTable(header=['delta_lnL'], rows = LRs)
>>> table.writeToFile("../results/tf_lnL_worse.txt", sep='\t')
```

#### Previous topic

Parameters estimated from TF models are biased by composition

#### Next topic

Filtering masked alignments

### This Page

- Show Source

### Quick search

### Navigation

- index
- modules |
- next |
- previous |
- Context Dependent Substitutions v-Draft documentation »

© Copyright 2008, Gavin Huttley.
Last updated on Sep 24, 2008.
Created using Sphinx.
