# Supplementary material for: Pitfalls of the most commonly used models of context dependent substitution
Source: Biol Direct. 2008 Dec 16;3:52. doi: 10.1186/1745-6150-3-52 (PMC2628887; doi:10.1186/1745-6150-3-52)
Supplement: Additional file 2 — Scripts used in the study. Archive of stand-alone web site presenting the central scripts used in this study. [file 1745-6150-3-52-S2.zip › HuttleyAdditional2/index.html]

Scripts: Context Dependent Substitution — Context Dependent Substitutions v-Draft documentation


### Navigation

- index
- modules |
- next |
- Context Dependent Substitutions v-Draft documentation »

# Scripts: Context Dependent Substitution¶

This document is a companion to a manuscript contrasting the properties of two independent-tuple context dependent substitution model forms. The two model forms differ in their weighting of the instantaneous rate matrix, employing either nucleotide frequencies or tuple frequencies and are referred to as the NF and TF forms respectively.

The purpose of this document is to provide a more readable form of the key scripts used in this study with some additional commentary. An archive of the actual scripts and data generated/used in the study is also included as a companion directory. Running the analysis scripts requires installation of the modified version of PyCogent (distributed as Additional data file 1) and its dependencies. Running the visualisation and summary scripts requires matplotlib (version 0.98 or greater).

Data used in the study, both simulated and real sequence alignments, and the raw Python scripts are available on request from the corresponding author.

The order of content listing matches the order of dicussion in the main manuscript.

**Contents:**

- Parameters estimated from TF models are biased by composition

- The likelihood of the TF form can be worse than that of a comparble nucleotide model

- Filtering masked alignments

- Fitting the dinucleotide GTR model

- Fitting individual dinucleotide parameters

# Indices and tables¶

- *Search Page*

### Table Of Contents

- Scripts: Context Dependent Substitution
- Indices and tables

#### Next topic

Parameters estimated from TF models are biased by composition

### This Page

- Show Source

### Quick search

### Navigation

- index
- modules |
- next |
- Context Dependent Substitutions v-Draft documentation »

© Copyright 2008, Gavin Huttley.
Last updated on Oct 02, 2008.
Created using Sphinx.
