# Supplementary material for: Pitfalls of the most commonly used models of context dependent substitution
Source: Biol Direct. 2008 Dec 16;3:52. doi: 10.1186/1745-6150-3-52 (PMC2628887; doi:10.1186/1745-6150-3-52)
Supplement: Additional file 2 — Scripts used in the study. Archive of stand-alone web site presenting the central scripts used in this study. [file 1745-6150-3-52-S2.zip › HuttleyAdditional2/modindex.html]

Global Module Index — Context Dependent Substitutions v-Draft documentation


### Navigation

- index
- modules |
- Context Dependent Substitutions v-Draft documentation »

# Global Module Index

---

### Quick search

### Navigation

- index
- modules |
- Context Dependent Substitutions v-Draft documentation »

© Copyright 2008, Gavin Huttley.
Last updated on Oct 02, 2008.
Created using Sphinx.
