# Supplementary material for: Pitfalls of the most commonly used models of context dependent substitution
Source: Biol Direct. 2008 Dec 16;3:52. doi: 10.1186/1745-6150-3-52 (PMC2628887; doi:10.1186/1745-6150-3-52)
Supplement: Additional file 1 — Modified version of PyCogent library. Archive of the PyCogent library developed for this study from PyCogent version 1.1. [file 1745-6150-3-52-S1.zip › HuttleyAdditional1/doc/index.html]

xml version="1.0" encoding="utf-8" ?


Cogent Usage Examples


# Cogent Usage Examples

Contents

- The Readme
  - COGENT (COmparative GENomics Toolkit) Install
    - Dependencies
    - Installation
    - Testing
    - Tips for usage
    - Citation
    - Licenses and disclaimer
    - Contacts
- A Note on the Computable Documentation
- Data manipulation
  - Translating DNA into protein
  - Advanced sequence handling
  - Getting the reverse complement
  - Map protein alignment gaps to DNA alignment gaps
- Data Visualisation
  - Drawing dendrograms and saving to PDF
  - Drawing a dotplot
- Modelling Evolution
  - The simplest script
  - Performing a relative rate test
  - A test of the neutral theory
  - Use an empirical protein substitution model
  - Analysis of rate heterogeneity
  - Likelihood analysis of multiple loci
  - Reusing results to speed up optimisation
  - Specifying and using an restricted nucleotide substitution model
  - Simulate an alignment
  - Performing a parametric bootstrap
  - Estimate parameter values using a sampling from a dataset
- Phylogenetic Reconstruction
  - Calculate pairwise distances between sequences
  - Make a neighbor joining tree
  - Phylogenetic reconstruction by least squares
    - Look for the single best tree
    - A more rigourous tree space search
    - Assessing the fit for a pre-specified tree topology
    - Using maximum likelihood for measuring tree fit
  - Making a phylogenetic tree from a protein sequence alignment
- Python Coding Guidelines
  - Why have coding guidelines?
    - What should I call my variables?
      - Acceptable abbreviations
    - What are the naming conventions?
    - How do I organize my modules (source files)?
      - Example of module structure
      - How should I write comments?
    - How should I format my code?
    - How should I test my code ?
      - Some unittest pointers
      - Example of a unittest test module structure

# The Readme

## COGENT (COmparative GENomics Toolkit) Install

|  |  |
| --- | --- |
| Author: | Gavin Huttley, Rob Knight |
| Address: | John Curtin School of Medical Research, Australian National University, Canberra, ACT 0200, Australia. Department of Chemistry and Biochemistry, University of Colorado, Boulder, CO 80309-0215, USA. |
| Version: | 1.0.1 |
| Copyright: | This software is copyright 2002-2007. |
| Download: | Download from here. |
| Registration: | To be informed of bugs, new releases please subscribe to the mailing lists at sourceforge. |

### Dependencies

The toolkit requires Python 2.5.1 or greater, and Numpy 1.0 or greater. Aside from these the dependencies below are optional and the code will work as is. A C compiler, however, will allow external C module's responsible for the likelihood and matrix exponentiation calculations to be compiled, resulting in significantly improved performance.

**For non-geeks:** The best way to install these dependencies depends on your platform. For MacOS X users, we suggest you install MacPython (see the python home page) as it also contains a package manager that can ease installation of some of the dependencies.

Required
:   - Python: the language the toolkit is primarily written in, and in which the user writes control scripts.
    - Numpy: This is a python module used for speeding up matrix computations. It is available as source code for \*nix. **NOTE:** For installing Numpy, unless you know what you are doing we recommend not linking Numpy against ATLAS or LAPACK libraries. On systems where these have been installed, this can be achieved by setting shell environment variables prior to building Numpy as:

      ```
      $ export ATLAS=None
      $ export LAPACK=None
      ```

Optional
:   - C compiler: This is standard on most \*nix platforms. On Macos X this is available for free in the Developer tools which, if you don't already have them, can be obtained from Apple, or get MacPython and use the package manager.
    - ReportLab: required for drawing trees and alignments to pdf.
    - Matplotlib: used to plot several kinds of graphs related to codon usage.
    - Pyrex: This module is only necessary if you are a developer who wants to modify the \*.pyx files.
    - PyxMPI: Our own python MPI interface, required for parallel computation.

### Installation

For \*nix platforms (including MacOS X), installation of the software is conventional for python packages. Download the software from here. Uncompress the archive and change into the Cogent directory and type:

```
$ python setup.py build
```

This automatically compiles the modules. If you have administrative privileges type:

```
$ sudo python setup.py install
```

This then places the entire package into your python/site-packages folder.

If you do not have administrator privileges on your machine you can move the cogent directory to where you want it (or leave it in place) and add this location to your python path using sys.path.append("/your/path/to/Cogent") in each script, or by setting shell environment variables. (Note that the path is not to ../Cogent/cogent.)

### Testing

Cogent/tests contains all the tests (currently >2700). You can most readily run the tests using the Cogent/run\_tests shell script. This is done by typing:

```
$ sh run_tests
```

which will automatically build extensions in place, set up the PYTHONPATH and run Cogent/tests/alltests.py. Note that if certain optional applications are not installed this will be indicated in the output as "can't find" or "not installed". A . will be printed to screen for each test and if they all pass, you'll see output like:

Ran 1982 tests in 40.455s

OK

### Tips for usage

A good IDE can greatly simplify writing control scripts. Features such as code completion and definition look-up are extremely useful. Amongst the freeware editors are Jedit (runs on all platforms, and has plugins to provide an overview of code structure or to organise projects), SubEthaEdit (MacOS X only) and of course emacs (all platforms). These provide syntax highlighting for Python, automated code indentation and (Jedit and emacs) code-folding abilities. For a more complete list of editors go here.

To get help on attributes of an object in python, use the dir(myalign) to list the attributes of myalign or help(myalign.writeToFile) to figure out how to use the myalign.writeToFile method. Also note that the directory structure of the package is similar to the import statements required to use a module -- to see the contents of alignment.py or sequence.py you need to look in the directory cogent/core path, to use the classes in those files you specify cogent.core for importing.

### Citation

If you use this software for published work please cite either -- R. Knight, P. Maxwell, A. Birmingham, J. Carnes, J. Caporaso, B. Easton, M. Hamady, H. Lindsay, Z. Liu, C. Lozupone, R. Sammut, S. Smit, M. Wakefield, J. Widmann, S. Wikman, S. Wilson, H. Ying, and G. Huttley. PyCogent: a toolkit for making sense from sequence. Genome Biol, In press, 2007.; or, Butterfield, A., V. Vedagiri, E. Lang, C. Lawrence, M.J. Wakefield, A. Isaev, and G.A. Huttley, PyEvolve: a toolkit for statistical modelling of molecular evolution. BMC Bioinformatics, 2004. **5(1):** p. 1

### Licenses and disclaimer

COGENT is released under the GPL license, a copy of which is included in the distribution. A copy of the permission to use the matrix exponentiation code from PAML is also included. Licenses for other code sources are left in place.

This software is provided "as-is". There are no expressed or implied warranties of any kind, including, but not limited to, the warranties of merchantability and fitness for a given application. In no event shall the authors be liable for any direct, indirect, incidental, special, exemplary or consequential damages (including, but not limited to, loss of use, data or profits, or business interruption) however caused and on any theory of liability, whether in contract, strict liability or tort (including negligence or otherwise) arising in any way out of the use of this software, even if advised of the possibility of such damage.

### Contacts

If you find a bug or have any questions please send us an email.

# A Note on the Computable Documentation

The following examples are all available as standalone text files which can be computed using the Python doctest module. One caveat with these tests is a subset will fail sporadically (or even consistently), although there is nothing 'wrong' with the software. These failures arise because of the typically very small data-sets we use in order for the documentation to compute in a short time period. As a result of their small size, the results from numerical optimisations are volatile and can change from one run to another -- leading to 'failures'. Specific examples that are prone to these problems involve the HMM models, the test of Neutrality, rate heterogeneity, unrestricted nucleotide substitution model and even the simplest example.

# Data manipulation

## Translating DNA into protein

To translate a DNA alignment, read it in assigning the DNA alphabet. Note setting aligned = False is critical for loading sequences of unequal length. Different genetic codes are available in cogent.core.genetic\_code

```
>>> from cogent import LoadSeqs, DNA
>>> al = LoadSeqs('data/test2.fasta', moltype=DNA, aligned = False)
>>> pal = al.getTranslation()
>>> print pal.toFasta()
>DogFaced
ARSQQNRWVETKETCNDRQT
>HowlerMon
ARSQHNRWAESEETCNDRQT
>Human
ARSQHNRWAGSKETCNDRRT
>Mouse
AVSQQSRWAASKGTCNDRQV
>NineBande
RQQSRWAESKETCNDRQT
```

To save this result to a file, use the writeToFile method.

## Advanced sequence handling

Individual sequences and alignments can be manipulated by annotations. Most value in the genome sequences arises from sequence annotations regarding specific sequence feature types, e.g. genes with introns / exons, repeat sequences. These can be applied to an alignment either using data formats available from genome portals (e.g. GFF, or GenBank annotation formats) or by custom assignments.

Annotations can be added in two ways: using either the addAnnotation or the addFeature method. The distinction between these two is that addFeatures is more specialised. Features can be thought of as a type of annotation representing standard sequence properties eg introns/exons. Annotations are the more general case, such as a computed property which has, say a numerical value and a span.

For illustrative purposes we define a sequence with 2 exons and grab the 1^st^ exon:

```
>>> from cogent import DNA
>>> s = DNA.makeSequence("aagaagaagacccccaaaaaaaaaattttttttttaaaaaaaaaaaaa",
... Name="Orig")
>>> exon1 = s.addFeature('exon', 'exon1', [(10,15)])
>>> exon2 = s.addFeature('exon', 'exon2', [(30,40)])
```

Here, 'exon' is the feature type, and 'exon#' the feature name. The feature type is used for the display formatting, which won't be illustrated here, and also for selecting all features of the same type, shown below.

We could also have created an annotation using the addAnnotation method:

```
>>> from cogent.core.annotation import Feature
>>> s2=DNA.makeSequence("aagaagaagacccccaaaaaaaaaattttttttttaaaaaaaaaaaaa",
... Name="Orig2")
>>> exon3 = s2.addAnnotation(Feature, 'exon', 'exon1', [(35,40)])
```

We can use the features (eg exon1) to get the corresponding sequence region.

```
>>> s[exon1]
DnaSequence(CCCCC)
```

You can query annotations by type and optionally by label, receiving a list of features:

```
>>> exons = s.getAnnotationsMatching('exon')
>>> print exons
[exon "exon1" at [10:15]/48, exon "exon2" at [30:40]/48]
```

We can use this list to construct a pseudo-feature covering (or excluding) multiple features using getRegionCoveringAll. For instance, getting all exons,

```
>>> print s.getRegionCoveringAll(exons)
region "exon" at [10:15, 30:40]/48
>>> s.getRegionCoveringAll(exons).getSlice()
DnaSequence(CCCCCTT... 15)
```

or not exons (the exon *shadow*):

```
>>> print s.getRegionCoveringAll(exons).getShadow().getSlice()
AAGAAGAAGAAAAAAAAAAATTTTTAAAAAAAA
```

The first of these essentially returns the CDS of the gene.

Features are themselves sliceable:

```
>>> exon1[0:3].getSlice()
DnaSequence(CCC)
```

This approach to sequence / alignment handling allows the user to manipulate them according to things they know about such as genes or repeat elements. Most of this annotation data can be obtained from genome portals.

The toolkit can perform standard sequence / alignment manipulations such as getting a subset of sequences or aligned columns, translating sequences, reading and writing standard formats.

## Getting the reverse complement

This is a property of DNA, and hence alignments need to be created with the appropriate MolType. In the following example, the alignment is truncated to just 100 bases for the sake of simplifying the presentation.

```
>>> from cogent import LoadSeqs, DNA
>>> aln = LoadSeqs("data/long_testseqs.fasta", moltype=DNA)[:100]
```

The original alignment looks like this.

```
>>> print aln
>FlyingFox
TGTGGCACAAATGCTCATGCCAGCTCTTTACAGCATGAGAAC---AGTTTATTATACACTAAAGACAGAATGAATGTAGAAAAGACTGACTTCTGTAATA
>DogFaced
TGTGGCACAAATACTCATGCCAACTCATTACAGCATGAGAACAGCAGTTTATTATACACTAAAGACAGAATGAATGTAGAAAAGACTGACTTCTGTAATA
```

We do reverse complement very simply.

```
>>> naln = aln.rc()
```

The reverse complemented alignment looks like this.

```
>>> print naln
>FlyingFox
TATTACAGAAGTCAGTCTTTTCTACATTCATTCTGTCTTTAGTGTATAATAAACT---GTTCTCATGCTGTAAAGAGCTGGCATGAGCATTTGTGCCACA
>DogFaced
TATTACAGAAGTCAGTCTTTTCTACATTCATTCTGTCTTTAGTGTATAATAAACTGCTGTTCTCATGCTGTAATGAGTTGGCATGAGTATTTGTGCCACA
<BLANKLINE>
```

## Map protein alignment gaps to DNA alignment gaps

Although PyCogent provides a means for directly aligning codon sequences, you may want to use a different approach based on the translate-align-introduce gaps into the original paradigm. After you've translated your codon sequences, and aligned the resulting amino acid sequences, you want to introduce the gaps from the aligned protein sequences back into the original codon sequences. Here's how.

```
>>> from cogent import LoadSeqs, DNA, PROTEIN
```

First I'm going to construct an artificial example, using the seqs dict as a means to get the data into the Alignment object. The basic idea, however, is that you should already have a set of DNA sequences that are in frame (i.e. position 0 is the 1st codon position), you've translated those sequences and aligned these translated sequences. The result is an alignment of aa sequences and a set of unaligned DNA sequences from which the aa seqs were derived. If your sequences are not in frame you can adjust it by either slicing, or adding N's to the beginning of the raw string.

```
>>> seqs = {
... 'hum': 'AAGCAGATCCAGGAAAGCAGCGAGAATGGCAGCCTGGCCGCGCGCCAGGAGAGGCAGGCCCAGGTCAACCTCACT',
... 'mus': 'AAGCAGATCCAGGAGAGCGGCGAGAGCGGCAGCCTGGCCGCGCGGCAGGAGAGGCAGGCCCAAGTCAACCTCACG',
... 'rat': 'CTGAACAAGCAGCCACTTTCAAACAAGAAA'}
>>> unaligned_DNA = LoadSeqs(data=seqs, moltype = DNA, aligned = False)
>>> print unaligned_DNA.toFasta()
>hum
AAGCAGATCCAGGAAAGCAGCGAGAATGGCAGCCTGGCCGCGCGCCAGGAGAGGCAGGCCCAGGTCAACCTCACT
>mus
AAGCAGATCCAGGAGAGCGGCGAGAGCGGCAGCCTGGCCGCGCGGCAGGAGAGGCAGGCCCAAGTCAACCTCACG
>rat
CTGAACAAGCAGCCACTTTCAAACAAGAAA
```

In order to ensure the alignment algorithm preserves the coding frame, we align the translation of the sequences. We need to translate them first, but note that because the seqs are unaligned they we have to set aligned = False, or we'll get an error.

```
>>> unaligned_aa = unaligned_DNA.getTranslation()
>>> print unaligned_aa.toFasta()
>hum
KQIQESSENGSLAARQERQAQVNLT
>mus
KQIQESGESGSLAARQERQAQVNLT
>rat
LNKQPLSNKK
```

The translated seqs can then be written to file, using the method writeToFile. That file then serves as input for an alignment program. The resulting alignment file can be read back in. (We won't write to file in this example.) For this example we will specify the aligned sequences in the dict, rather than from file.

```
>>> aligned_aa_seqs = {'hum': 'KQIQESSENGSLAARQERQAQVNLT',
... 'mus': 'KQIQESGESGSLAARQERQAQVNLT',
... 'rat': 'LNKQ------PLS---------NKK'}
>>> aligned_aa = LoadSeqs(data = aligned_aa_seqs, moltype = PROTEIN)
>>> aligned_DNA = aligned_aa.replaceSequences(unaligned_DNA)
```

Just to be sure, we'll check that the DNA sequence has gaps in the right place.

```
>>> print aligned_DNA
>hum
AAGCAGATCCAGGAAAGCAGCGAGAATGGCAGCCTGGCCGCGCGCCAGGAGAGGCAGGCCCAGGTCAACCTCACT
>rat
CTGAACAAGCAG------------------CCACTTTCA---------------------------AACAAGAAA
>mus
AAGCAGATCCAGGAGAGCGGCGAGAGCGGCAGCCTGGCCGCGCGGCAGGAGAGGCAGGCCCAAGTCAACCTCACG
<BLANKLINE>
```

# Data Visualisation

## Drawing dendrograms and saving to PDF

From cogent import all the components we need.

```
>>> from cogent import LoadSeqs, LoadTree
>>> from cogent.evolve.models import Y98
>>> from cogent.draw import dendrogram
```

Do a model, see the neutral test example for more details of this

```
>>> al = LoadSeqs("data/test.paml")
>>> t = LoadTree("data/test.tree")
>>> sm = Y98()
>>> nonneutral_lf = sm.makeLikelihoodFunction(t)
>>> nonneutral_lf.setParamRule("omega", is_independent = 1)
>>> nonneutral_lf.setAlignment(al)
>>> nonneutral_lf.optimise(tolerance = 1.0)
Outer loop = 0...
>>> nonneutral_lf.optimise(local = True)
    Number of function evaluations = 1; current F = 139...
```

We will draw two different dendrograms -- one with branch lengths contemporaneous, the other where length is scaled NOTE: the argument names to dendrogram classes break from our convention of underscores\_separating\_argument\_words. The convention used is that of ReportLab and these key-word arguments are passed directly to the underlying reportlab module.

Specify the dimensions of the canvas in pixels

```
>>> height, width = 500, 700
```

Dendrogram with branch lengths not proportional

```
>>> np = dendrogram.ContemporaneousDendrogram(nonneutral_lf.tree)
>>> np.drawToPDF('tree-unscaled.pdf' , width, height, stroke_width=2.0,
... show_params = ['r'], label_template = "%(r).2g", shade_param = 'r',
... max_value = 1.0, show_internal_labels=False, font_size = 10,
... scale_bar = None, use_lengths=False)
```

Dendrogram with branch lengths proportional

```
>>> p = dendrogram.SquareDendrogram(nonneutral_lf.tree)
>>> p.drawToPDF('tree-scaled.pdf', width, height, stroke_width=2.0,
... shade_param = 'r', max_value = 1.0, show_internal_labels=False,
... font_size = 10)
```

To save a tree for later reuse, either for analysis of drawing can be done using an annotated tree, which looks just like a tree, but has the maximum-likelihood parameter estimates attached to each tree edge. This tree can be saved in xml format, which preserve these parameter estimates. The annotated tree is obtained from the likelihood function with following command.

```
>>> at = nonneutral_lf.getAnnotatedTree()
```

Saving this to file is done using the normal writeToFile method, specifying a filename with the .xml suffix.

## Drawing a dotplot

```
>>> from cogent import LoadSeqs
>>> from cogent.core import annotation
>>> from cogent.draw import dotplot
```

Load the alignment for illustrative purposes, I'll make one sequence a different length than the other and introduce a custom sequence annotation for a miscellaneous feature. Normally, those annotations would be on the unaligned sequences.

```
>>> aln = LoadSeqs("data/test.paml")
>>> feature = aln.addAnnotation(annotation.Feature, "misc_feature",
...                             "pprobs", [(38, 55)])
>>> seq1 = aln.getSeq('NineBande')[10:-3]
>>> seq2 = aln.getSeq('DogFaced')
```

Write out the dotplot as a pdf file in the current directory note that seq1 will be the x-axis, and seq2 the y-axis.

```
>>> dp = dotplot.Display2D(seq1,seq2)
>>> filename = 'dotplot_example.pdf'
>>> dp.drawToPDF(filename)
```

# Modelling Evolution

## The simplest script

This is just about the simplest possible Cogent script. We use a canned nucleotide substitution model: the general time reversible model.

```
>>> from cogent.evolve.models import GTR
>>> from cogent import LoadSeqs, LoadTree
>>> model = GTR()
>>> alignment = LoadSeqs("data/test.paml")
>>> tree = LoadTree("data/test.tree")
>>> likelihood_function = model.makeLikelihoodFunction(tree)
>>> likelihood_function.setAlignment(alignment)
>>> likelihood_function.optimise(show_progress = False)
>>> print likelihood_function
Likelihood Function Table
==============================================
   A/C       A/G       A/T       C/G       C/T
----------------------------------------------
0.7120    2.1574    0.0000    0.4457    4.1764
----------------------------------------------
=============================
     edge    parent    length
-----------------------------
    Human    edge.0    0.0348
HowlerMon    edge.0    0.0168
   edge.0    edge.1    0.0222
    Mouse    edge.1    0.2047
   edge.1      root    0.0000
NineBande      root    0.0325
 DogFaced      root    0.0554
-----------------------------
===============
motif    mprobs
---------------
    T    0.1433
    C    0.1600
    A    0.3800
    G    0.3167
---------------
```

## Performing a relative rate test

From cogent import all the components we need

```
>>> from cogent import LoadSeqs, LoadTree
>>> from cogent.evolve.models import HKY85
>>> from cogent.maths import stats
```

Get your alignment and tree.

```
>>> al = LoadSeqs(filename = "data/test.paml")
>>> t = LoadTree(filename = "data/test.tree")
```

Create a HKY85 model.

```
>>> sm = HKY85()
```

Make the controller object.

```
>>> lf = sm.makeLikelihoodFunction(t)
```

Set the local clock for humans & Howler Monkey. This method is just a special interface to the more general setParamRules method.

```
>>> lf.setLocalClock("Human", "HowlerMon")
```

Get the likelihood function object this object performs the actual likelihood calculation.

```
>>> lf.setAlignment(al)
```

Optimise the function capturing the return optimised lnL, and parameter value vector.

```
>>> lf.optimise(show_progress = False)
```

View the resulting maximum-likelihood parameter values.

```
>>> lf.setName("clock")
>>> print lf
clock
======
 kappa
------
4.8020
------
=============================
     edge    parent    length
-----------------------------
    Human    edge.0    0.0257
HowlerMon    edge.0    0.0257
   edge.0    edge.1    0.0224
    Mouse    edge.1    0.2112
   edge.1      root    0.0000
NineBande      root    0.0327
 DogFaced      root    0.0545
-----------------------------
===============
motif    mprobs
---------------
    T    0.1433
    C    0.1600
    A    0.3800
    G    0.3167
---------------
```

We extract the log-likelihood and number of free parameters for later use.

```
>>> null_lnL = lf.getLogLikelihood()
>>> null_nfp = lf.getNumFreeParams()
```

Clear the local clock constraint, freeing up the branch lengths.

```
>>> lf.setParamRule('length', is_independent=True)
```

Run the optimiser capturing the return optimised lnL, and parameter value vector.

```
>>> lf.optimise(show_progress=False)
```

View the resulting maximum-likelihood parameter values.

```
>>> lf.setName("non clock")
>>> print lf
non clock
======
 kappa
------
4.8027
------
=============================
     edge    parent    length
-----------------------------
    Human    edge.0    0.0347
HowlerMon    edge.0    0.0167
   edge.0    edge.1    0.0224
    Mouse    edge.1    0.2112
   edge.1      root    0.0000
NineBande      root    0.0327
 DogFaced      root    0.0545
-----------------------------
===============
motif    mprobs
---------------
    T    0.1433
    C    0.1600
    A    0.3800
    G    0.3167
---------------
```

These two lnL's are now used to calculate the likelihood ratio statistic it's degrees-of-freedom and the probability of observing the LR.

```
>>> LR = 2 * (lf.getLogLikelihood() - null_lnL)
>>> df = lf.getNumFreeParams() - null_nfp
>>> P = stats.chisqprob(LR, df)
```

Print this and look up a chi-sq with number of edges - 1 degrees of freedom.

```
>>> print "Likelihood ratio statistic = ", LR
Likelihood ratio statistic =  0.34...
>>> print "degrees-of-freedom = ", df
degrees-of-freedom =  1
>>> print "probability = ", P
probability =  0.5...
```

## A test of the neutral theory

This file contains an example for performing a likelihood ratio test of neutrality. The test compares a model where the codon model parameter omega is constrained to be the same for all edges against one where each edge has its' own omega. From cogent import all the components we need.

```
>>> from cogent import LoadSeqs, LoadTree
>>> from cogent.evolve.models import GY94
>>> from cogent.maths import stats
```

Get your alignment and tree.

```
>>> al = LoadSeqs("data/test.paml")
>>> t = LoadTree("data/test.tree")
```

We use a Goldman Yang 1994 model.

```
>>> sm = GY94()
```

Make the controller object

```
>>> lf = sm.makeLikelihoodFunction(t)
```

Get the likelihood function object this object performs the actual likelihood calculation.

```
>>> lf.setAlignment(al)
```

By default, parameters other than branch lengths are treated as global in scope, so we don't need to do anything special here. We can influence how rigorous the optimisation will be, and switch between the global and local optimisers provided in the toolkit using arguments to the optimise method. The global\_tolerance=1.0 argument specifies conditions for an early break from simulated annealing which will be automatically followed by the Powell local optimiser. :Note: the 'results' are of course nonsense.

```
>>> lf.optimise(global_tolerance = 1.0, show_progress=False)
```

View the resulting maximum-likelihood parameter values

```
>>> print lf
Likelihood Function Table
================
 kappa     omega
----------------
9.2738    1.8707
----------------
=============================
     edge    parent    length
-----------------------------
    Human    edge.0    0.0968
HowlerMon    edge.0    0.0540
   edge.0    edge.1    0.0654
    Mouse    edge.1    0.9115
   edge.1      root    0.0000
NineBande      root    0.1073
 DogFaced      root    0.1801...
```

We'll get the lnL and number of free parameters for later use.

```
>>> null_lnL = lf.getLogLikelihood()
>>> null_nfp = lf.getNumFreeParams()
```

Specify each edge has it's own omega by just modifying the existing lf. This means the new function will start with the above values.

```
>>> lf.setParamRule("omega", is_independent = True)
```

Optimise the likelihood function, this time just using the local optimiser.

```
>>> lf.optimise(local = True, show_progress=False)
```

View the resulting maximum-likelihood parameter values.

```
>>> print lf
Likelihood Function Table
======
 kappa
------
8.9536
------
============================================
     edge    parent    length          omega
--------------------------------------------
    Human    edge.0    0.0970    999999.9815
HowlerMon    edge.0    0.0569    999999.9370
   edge.0    edge.1    0.0700    999999.9867
    Mouse    edge.1    0.9602         0.6964
   edge.1      root    0.0000    196705.5616
NineBande      root    0.1114    999999.9940
 DogFaced      root    0.1809         1.0999...
```

|  |  |
| --- | --- |
| Note: | The parameter estimates for omega are highly implausible, reflecting (in this case) our small and uninformative data set. |

Get out an annotated tree, it looks just like a tree, but has the maximum-likelihood parameter estimates attached to each tree edge. This object can be used for plotting, or to provide starting estimates to a related model.

```
>>> at = lf.getAnnotatedTree()
```

Get a dictionary of the statistics that I could use for post-processing.

```
>>> sd = lf.getStatisticsAsDict(with_edge_names=True)
```

The lnL's from the two models are now used to calculate the likelihood ratio statistic (LR) it's degrees-of-freedom (df) and the probability (P) of observing the LR.

```
>>> LR = 2 * (lf.getLogLikelihood() - null_lnL)
>>> df = lf.getNumFreeParams() - null_nfp
>>> P = stats.chisqprob(LR, df)
```

Print this and look up a chi-sq with number of edges - 1 degrees of freedom.

```
>>> print "Likelihood ratio statistic = ", LR
Likelihood ratio statistic =  4.4...
>>> print "degrees-of-freedom = ", df
degrees-of-freedom =  6
>>> print "probability = ", P
probability =  0.6...
```

## Use an empirical protein substitution model

This file contains an example of importing an empirically determined protein substitution matrix such as Dayhoff et al 1978 and using it to create a substitution model. The globin alignment is from the PAML distribution.

```
>>> from cogent import LoadSeqs, LoadTree, PROTEIN
>>> from cogent.evolve.substitution_model import EmpiricalProteinMatrix
>>> from cogent.parse.paml_matrix import PamlMatrixParser
```

Make a tree object. In this case from a string.

```
>>> treestring="(((rabbit,rat),human),goat-cow,marsupial);"
>>> t = LoadTree(treestring=treestring)
```

Import the alignment, explicitly setting the moltype to be protein

```
>>> al = LoadSeqs('data/abglobin_aa.phylip',
...                interleaved=True,
...                moltype=PROTEIN,
...                )
```

Open the file that contains the empirical matrix and parse the matrix and frequencies.

```
>>> matrix_file = open('data/dayhoff.dat')
```

The PamlMatrixParser will import the matrix and frequency from files designed for Yang's PAML package. This format is the lower half of the matrix in three letter amino acid name order white space delineated followed by motif frequencies in the same order.

```
>>> empirical_matrix, empirical_frequencies = PamlMatrixParser(matrix_file)
```

Create an Empirical Protein Matrix Substitution model object. This will take the unscaled empirical matrix and use it and the motif frequencies to create a scaled Q matrix.

```
>>> sm = EmpiricalProteinMatrix(empirical_matrix, empirical_frequencies)
```

Make a parameter controller, likelihood function object and optimise.

```
>>> lf = sm.makeLikelihoodFunction(t)
>>> lf.setAlignment(al)
>>> lf.optimise(show_progress = False)
>>> print lf.getLogLikelihood()
-1706...
>>> print lf
Likelihood Function Table
=============================
     edge    parent    length
-----------------------------
   rabbit    edge.0    0.0785
      rat    edge.0    0.1750
   edge.0    edge.1    0.0324
    human    edge.1    0.0545
   edge.1      root    0.0269
 goat-cow      root    0.0972
marsupial      root    0.2424
-----------------------------
===============
motif    mprobs
---------------
    A    0.0871
    C    0.0335
    D    0.0469
    E    0.0495
    F    0.0398
    G    0.0886
    H    0.0336
    I    0.0369
    K    0.0805
    L    0.0854
    M    0.0148
    N    0.0404
    P    0.0507
    Q    0.0383
    R    0.0409
    S    0.0696
    T    0.0585
    V    0.0647
    W    0.0105
    Y    0.0299
---------------
```

## Analysis of rate heterogeneity

A simple example for analyses involving rate heterogeneity among sites. In this case we will simulate an alignment with two rate categories and then try to recover the rates from the alignment.

```
>>> from cogent.evolve.substitution_model import Nucleotide
>>> from cogent import LoadTree
```

Make an alignment with equal split between rates 0.6 and 0.2, and then concatenate them to create a new alignment.

```
>>> model = Nucleotide(equal_motif_probs=True)
>>> tree = LoadTree("data/test.tree")
>>> lf = model.makeLikelihoodFunction(tree)
>>> lf.setParamRule('length', value=0.6, is_const=True)
>>> aln1 = lf.simulateAlignment(sequence_length=1000)
>>> lf.setParamRule('length', value=0.2, is_const=True)
>>> aln2 = lf.simulateAlignment(sequence_length=1000)
>>> aln3 = aln1 + aln2
```

Start from scratch, optimising only rates and the rate probability ratio.

```
>>> model = Nucleotide(equal_motif_probs=True, ordered_param="rate",
...                    distribution="free")
>>> lf = model.makeLikelihoodFunction(tree, bins=2)
>>> lf.setAlignment(aln3)
>>> lf.optimise(local=True, max_restarts=2, show_progress = False)
```

We want to know the bin probabilities and the posterior probabilities.

```
>>> bprobs = lf.getParamValue('bprobs')
>>> pp = lf.getBinProbs()
>>> for bin in [0,1]:
...     p = pp[bin]
...     rate = lf.getParamValue('rate', bin='bin%s'%bin)
...     print '%.2f of sites have rate %.2f' % (bprobs[bin], rate)
...     print 'Avg probs over the fast (%.2f) and slow (%.2f) halves' % \
...        (sum(p[:1000])/1000, sum(p[1000:])/1000)
0.12 of sites have rate 0.22
Avg probs over the fast (0.05) and slow (0.18) halves
0.88 of sites have rate 1.10
Avg probs over the fast (0.95) and slow (0.82) halves
```

We'll now use a gamma distribution on the sample alignment, specifying the number of bins as 4. We specify that the bins have equal density using the lf.setParamRule('bprobs', is\_const=True) command.

```
>>> model = Nucleotide(equal_motif_probs=True, ordered_param="rate",
...                    distribution="gamma")
>>> lf = model.makeLikelihoodFunction(tree, bins=4)
>>> lf.setParamRule('bprobs', is_const=True)
>>> lf.setAlignment(aln3)
>>> lf.optimise(local=True, max_restarts=2, show_progress = False)
```

## Likelihood analysis of multiple loci

We want to know whether an exchangeability parameter is different between alignments. We will specify a null model, under which each alignment get's it's own motif probabilities and all alignments share branch lengths and the exchangeability parameter kappa (the transition / transversion ratio). We'll split the example alignment into two-pieces.

```
>>> from cogent import LoadSeqs, LoadTree
>>> from cogent.evolve.models import HKY85
>>> from cogent.recalculation.scope import EACH, ALL
>>> from cogent.maths.stats import chisqprob
>>> aln = LoadSeqs("data/long_testseqs.fasta")
>>> half = len(aln)/2
>>> aln1 = aln[:half]
>>> aln2 = aln[half:]
```

We provide names for those alignments, then construct the tree, model instances.

```
>>> loci_names = ["1st-half", "2nd-half"]
>>> loci = [aln1, aln2]
>>> tree = LoadTree(tip_names=aln.getSeqNames())
>>> mod = HKY85()
```

To make a likelihood function with multiple alignments we provide the list of loci names. We can then specify a parameter (other than length) to be the same across the loci (using the imported ALL) or different for each locus (using EACH). We conduct a LR test as before.

```
>>> lf = mod.makeLikelihoodFunction(tree, loci=loci_names)
>>> lf.setParamRule("length", is_independent=False)
>>> lf.setParamRule('kappa', loci = ALL)
>>> lf.setAlignment(loci)
>>> lf.optimise(local=True, show_progress=False)
>>> print lf
Likelihood Function Table
===========================
  locus    motif    mprobs
---------------------------
1st-half        T    0.2341
1st-half        C    0.1758
1st-half        A    0.3956
1st-half        G    0.1944
2nd-half        T    0.2400
2nd-half        C    0.1851
2nd-half        A    0.3628
2nd-half        G    0.2121
---------------------------
================
kappa    length
----------------
8.0072    0.0271
----------------
>>> all_lnL = lf.getLogLikelihood()
>>> all_nfp = lf.getNumFreeParams()
>>> lf.setParamRule('kappa', loci = EACH)
>>> lf.optimise(local=True, show_progress=False)
>>> print lf
Likelihood Function Table
==================
   locus     kappa
------------------
1st-half    7.9077
2nd-half    8.1293
------------------
===========================
   locus    motif    mprobs
---------------------------
1st-half        T    0.2341
1st-half        C    0.1758
1st-half        A    0.3956
1st-half        G    0.1944
2nd-half        T    0.2400
2nd-half        C    0.1851
2nd-half        A    0.3628
2nd-half        G    0.2121
---------------------------
======
length
------
0.0271
------
>>> each_lnL = lf.getLogLikelihood()
>>> each_nfp = lf.getNumFreeParams()
>>> LR = 2 * (each_lnL - all_lnL)
>>> df = each_nfp - all_nfp
>>> print LR, df, chisqprob(LR, df)
0.00424532328725 1 0.94804967777
```

## Reusing results to speed up optimisation

An example of how to use the maximum-likelihood parameter estimates from one model as starting values for another model. In this file we do something silly, by saving a result and then reloading it. This is silly because the analyses are run consecutively. A better approach when running consecutively is to simply use the annotated tree directly.

```
>>> from cogent import LoadSeqs, LoadTree
>>> from cogent.evolve.models import Y98
```

We'll create a simple model, optimise it and save it for later reuse

```
>>> al = LoadSeqs("data/test.paml")
>>> t = LoadTree("data/test.tree")
>>> sm = Y98()
>>> lf = sm.makeLikelihoodFunction(t)
>>> lf.setAlignment(al)
>>> lf.optimise(local=True, show_progress=False)
>>> print lf
Likelihood Function Table
================
 kappa     omega
----------------
9.2759    1.8713
----------------
=============================
     edge    parent    length
-----------------------------
    Human    edge.0    0.0968
HowlerMon    edge.0    0.0540
   edge.0    edge.1    0.0654
    Mouse    edge.1    0.9116
   edge.1      root    0.0000
NineBande      root    0.1073
 DogFaced      root    0.1801...
```

The essential object for reuse is an annotated tree these capture the parameter estimates from the above optimisation we can either use this directly in the same run, or we can save the tree to file in xml format and reload the tree at a later time for use. In this example I'll illustrate the latter scenario.

```
>>> at=lf.getAnnotatedTree()
>>> at.writeToFile('tree.xml')
```

We load the tree as per usual

```
>>> nt = LoadTree('tree.xml')
```

Now create a more parameter rich model, in this case by allowing the Human edge to have a different value of omega. By providing the annotated tree, the parameter estimates from the above run will be used as starting values for the new model.

```
>>> new_lf = sm.makeLikelihoodFunction(nt)
>>> new_lf.setParamRule('omega', edge='Human',
... is_independent=True)
>>> new_lf.setAlignment(al)
>>> new_lf.optimise(local=True, show_progress=False)
>>> print new_lf
Likelihood Function Table
======
 kappa
------
9.0706
------
============================================
     edge    parent    length          omega
--------------------------------------------
    Human    edge.0    0.1001    999999.9965
HowlerMon    edge.0    0.0510         1.5666
   edge.0    edge.1    0.0649         1.5666
    Mouse    edge.1    0.8984         1.5666
   edge.1      root    0.0000         1.5666
NineBande      root    0.1064         1.5666
 DogFaced      root    0.1793         1.5666...
```

|  |  |
| --- | --- |
| Note: | A parameter rich model applied to a small data set is unreliable. |

## Specifying and using an restricted nucleotide substitution model

Do standard cogent imports.

```
>>> from cogent import LoadSeqs, LoadTree, DNA
>>> from cogent.evolve.predicate import MotifChange
>>> from cogent.evolve.substitution_model import Nucleotide
```

To specify substitution models we use the MotifChange class from predicates. In the case of an unrestricted nucleotide model, we specify 11 such MotifChanges, the last possible change being ignored (with the result it is constrained to equal 1, thus calibrating the matrix).

```
>>> ACTG = list('ACTG')
>>> preds = [MotifChange(i, j, forward_only=True) for i in ACTG for j in ACTG if i != j]
>>> del(preds[-1])
>>> preds
[A>C, A>T, A>G, C>A, C>T, C>G, T>A, T>C, T>G, G>A, G>C]
>>> sm = Nucleotide(predicates=preds, recode_gaps=True)
>>> print sm
<BLANKLINE>
Nucleotide ( name = ''; type = 'None'; params = ['A>T', 'C>G', 'T>G', 'G>A', 'T>A', 'T>C', 'C>A', 'G>C', 'C>T', 'A>G', 'A>C']; number of motifs = 4; motifs = ['T', 'C', 'A', 'G'])
<BLANKLINE>
```

We'll illustrate this with a sample alignment and tree in "data/test.paml".

```
>>> tr = LoadTree("data/test.tree")
>>> print tr
(((Human,HowlerMon),Mouse),NineBande,DogFaced);
>>> al = LoadSeqs("data/test.paml", moltype=DNA)
>>> al
5 x 60 dna alignment: NineBande[GCAAGGCGCCA...], Mouse[GCAGTGAGCCA...], Human[GCAAGGAGCCA...], ...
```

We now construct the parameter controller with each predicate constant across the tree, and get the likelihood function calculator.

```
>>> lf = sm.makeLikelihoodFunction(tr)
>>> lf.setAlignment(al)
>>> lf.setName('Unrestricted model')
>>> lf.optimise()
Outer loop = 0...
```

In the output from the optimise call you'll see progress from the simulated annealing optimiser which is used first, and the Powell optimiser which finishes things off.

```
>>> print lf
Unrestricted model
============================================================================
   A>C       A>G       A>T       C>A       C>G       C>T       G>A       G>C
----------------------------------------------------------------------------
0.6890    1.8880    0.0000    0.0000    0.0000    2.1652    0.2291    0.4868
----------------------------------------------------------------------------
<BLANKLINE>
continued:
====================================
   A>C       T>A       T>C       T>G
------------------------------------
0.6890    0.0000    2.2755    0.0000
------------------------------------
<BLANKLINE>
=============================
     edge    parent    length
-----------------------------
    Human    edge.0    0.0333
HowlerMon    edge.0    0.0165
   edge.0    edge.1    0.0164
    Mouse    edge.1    0.1980
   edge.1      root    0.0000
NineBande      root    0.0335
 DogFaced      root    0.0503
-----------------------------
===============
motif    mprobs
---------------
    T    0.1433
    C    0.1600
    A    0.3800
    G    0.3167
---------------
```

This data set is very small, so the parameter estimates are poor and hence doing something like allowing the parameters to differ between edges is silly. **But** if you have lots of data it makes sense and can be specified by modifying the lf as follows.

```
>>> for pred in preds:
...     lf.setParamRule(str(pred), is_independent=True)
```

You then make a new lf and optimise as above, but I won't do that now as the optimiser would struggle due to the low information content of this sample.

## Simulate an alignment

How to simulate an alignment. For this example we just create a simple model using a four taxon tree with very different branch lengths, a Felsenstein model with very different nucleotide frequencies and a long alignment.

See the other examples for how to define other substitution models.

```
>>> import sys
>>> from cogent import LoadTree
>>> from cogent.evolve import substitution_model
```

Specify the 4 taxon tree,

```
>>> t = LoadTree(treestring='(a:0.4,b:0.3,(c:0.15,d:0.2)edge.0:0.1);')
```

Define our Felsenstein 1981 substitution model.

```
>>> sm = substitution_model.Nucleotide(motif_probs = {'A': 0.5, 'C': 0.2,
... 'G': 0.2, 'T': 0.1}, model_gaps=False)
>>> lf = sm.makeLikelihoodFunction(t)
>>> lf.setConstantLengths()
>>> lf.setName('F81 model')
>>> print lf
F81 model
==========================
  edge    parent    length
--------------------------
     a      root    0.4000
     b      root    0.3000
     c    edge.0    0.1500
     d    edge.0    0.2000
edge.0      root    0.1000
--------------------------
===============
motif    mprobs
---------------
    T    0.1000
    C    0.2000
    A    0.5000
    G    0.2000
---------------
```

We'll now create a simulated alignment of length 1000 nucleotides.

```
>>> simulated = lf.simulateAlignment(sequence_length=1000)
```

The result is a normal Cogent alignment object, which can be used in the same way as any other alignment object.

## Performing a parametric bootstrap

This file contains an example for estimating the probability of a Likelihood ratio statistic obtained from a relative rate test. The bootstrap classes can take advantage of parallel architectures.

From cogent import all the components we need.

```
>>> from cogent import LoadSeqs, LoadTree
>>> from cogent.evolve import bootstrap
>>> from cogent.evolve.models import HKY85
>>> from cogent.maths import stats
```

Define the null model that takes an alignment object and returns a likelihood function properly assembled for optimising the likelihood under the null hypothesis. The sample distribution is generated using this model.

We will use a HKY model.

```
>>> def create_alt_function():
...     t = LoadTree("data/test.tree")
...     sm = HKY85()
...     return sm.makeLikelihoodFunction(t)
```

Define a function that takes an alignment object and returns an appropriately assembled function for the alternative model. Since the two models are identical bar the constraint on the branch lengths, we'll use the same code to generate the basic likelihood function as for the alt model, and then apply the constraint here

```
>>> def create_null_function():
...     lf = create_alt_function()
...     # set the local clock for humans & howler monkey
...     lf.setLocalClock("Human", "HowlerMon")
...     return lf
```

Get our observed data alignment

```
>>> aln = LoadSeqs(filename = "data/test.paml")
```

Create a EstimateProbability bootstrap instance

```
>>> estimateP = bootstrap.EstimateProbability(create_null_function(),
...                                       create_alt_function(),
...                                       aln)
```

Specify how many random samples we want it to generate. Here we use a very small number of replicates only for the purpose of testing.

```
>>> estimateP.setNumReplicates(5)
```

Run it.

```
>>> estimateP.run(show_progress = False)
```

show\_progress sets whether individual optimisations are printed to screen. Get the estimated probability.

```
>>> p = estimateP.getEstimatedProb()
```

p is a floating point value, as you'd expect. Grab the estimated likelihoods for the observed data.

```
>>> print '%.2f, %.2f' % estimateP.getObservedlnL()
-162.65, -162.48
```

## Estimate parameter values using a sampling from a dataset

This script uses the sample method of the alignment class to provide an
estimate for a two stage optimisation.
This allows rapid optimisation of long alignments and complex models with
a good chance of arriving at the global maximum for the model and data.
Local optimisation of the full alignment may end up in local maximum and
for this reason results from this strategy my be inaccurate.

From cogent import all the components we need.

```
>>> from cogent import LoadSeqs, LoadTree
>>> from cogent.evolve import  substitution_model
```

Load your alignment, note that if your file ends with a suffix that is the same as it's format (assuming it's a supported format) then you can just give the filename. Otherwise you can specify the format using the format argument.

```
>>> al = LoadSeqs(filename = "data/test.paml")
```

Get your tree

```
>>> t = LoadTree(filename = "data/test.tree")
```

Get the raw substitution model

```
>>> sm = substitution_model.Nucleotide()
```

Make a likelihood function from a sample of the alignment the .sample method selects the chosen number of bases at random because we set motif probabilities earlier the motif probabilities of the whole alignment rather than the sample will be used for the calculator

```
>>> lf = sm.makeLikelihoodFunction(t)
>>> lf.setMotifProbsFromData(al)
>>> lf.setAlignment(al.sample(20))
```

Optimise with the slower but more accurate simulated annealing optimiser

```
>>> lf.optimise()
Outer loop = 0...
```

Next use the whole alignment

```
>>> lf.setAlignment(al)
```

and the faster Powell optimiser that will only find the best result near the provided starting point

```
>>> lf.optimise(local=True)
Number of function evaluations = 1; current F = ...
```

Print the result using print lf.

# Phylogenetic Reconstruction

## Calculate pairwise distances between sequences

An example of how to calculate the pairwise distances for a set of sequences.

```
>>> from cogent import LoadSeqs
>>> from cogent.phylo import distance
```

Import a substitution model (or create your own)

```
>>> from cogent.evolve.models import HKY85
```

Load my alignment

```
>>> al = LoadSeqs("data/test.paml")
```

Create a pairwise distances object with your alignment and substitution model

```
>>> d = distance.EstimateDistances(al, submodel= HKY85())
```

Printing d before execution shows its status.

```
>>> print d
=========================================================================
Seq1 \ Seq2    NineBande       Mouse       Human    HowlerMon    DogFaced
-------------------------------------------------------------------------
  NineBande            *    Not Done    Not Done     Not Done    Not Done
      Mouse     Not Done           *    Not Done     Not Done    Not Done
      Human     Not Done    Not Done           *     Not Done    Not Done
  HowlerMon     Not Done    Not Done    Not Done            *    Not Done
   DogFaced     Not Done    Not Done    Not Done     Not Done           *
-------------------------------------------------------------------------
```

Which in this case is to simply indicate nothing has been done.

```
>>> d.run(show_progress=False)
>>> print d
=====================================================================
Seq1 \ Seq2    NineBande     Mouse     Human    HowlerMon    DogFaced
---------------------------------------------------------------------
  NineBande            *    0.2196    0.0890       0.0700      0.0891
      Mouse       0.2196         *    0.2737       0.2736      0.2467
      Human       0.0890    0.2737         *       0.0530      0.1092
  HowlerMon       0.0700    0.2736    0.0530            *      0.0894
   DogFaced       0.0891    0.2467    0.1092       0.0894           *
---------------------------------------------------------------------
```

Note that pairwise distances can be distributed for computation across multiple CPU's. In this case, when statistics (like distances) are requested only the master CPU returns data.

We'll write a phylip formatted distance matrix.

```
>>> d.writeToFile('junk.phylip', format="phylip")
```

We'll also save the distances to file in Python's pickle format.

```
>>> import cPickle
>>> f = open('dists_for_phylo.pickle', "w")
>>> cPickle.dump(d.getPairwiseDistances(), f)
>>> f.close()
```

## Make a neighbor joining tree

An example of how to calculate the pairwise distances for a set of sequences.

```
>>> from cogent import LoadSeqs
>>> from cogent.phylo import distance, nj
```

Import a substitution model (or create your own)

```
>>> from cogent.evolve.models import HKY85
```

Load the alignment.

```
>>> al = LoadSeqs("data/test.paml")
```

Create a pairwise distances object calculator for the alignment, providing a substitution model instance.

```
>>> d = distance.EstimateDistances(al, submodel= HKY85())
>>> d.run(show_progress=False)
```

Now use this matrix to build a neighbour joining tree.

```
>>> mytree = nj.nj(d.getPairwiseDistances())
>>> print mytree.asciiArt()
                    /-NineBande
          /edge.1--|
         |          \-Mouse
         |
-root----|--DogFaced
         |
         |          /-HowlerMon
          \edge.0--|
                    \-Human
```

We can save this tree to file.

```
>>> mytree.writeToFile('test_nj.tree')
```

## Phylogenetic reconstruction by least squares

We will load some pre-computed pairwise distance data. To see how that data was computed see the calculating pairwise distances example. That data is saved in a format called pickle which is native to python. As per usual, we import the basic components we need.

```
>>> import cPickle
>>> from cogent.phylo import distance, least_squares
```

Now load the distance data.

```
>>> filename = "dists_for_phylo.pickle"
>>> f = file(filename, 'r')
>>> dists = cPickle.load(f)
>>> f.close()
```

If there are extremely small distances, they can cause an error in the least squares calculation. Since such estimates are between extremely closely related sequences we could simply drop all distances for one of the sequences. We won't do that here, we'll leave that as exercise.

We make the ls calculator.

```
>>> ls = least_squares.WLS(dists)
```

We will search tree space for the collection of best trees using the advanced stepwise addition algorithm (hereafter *asaa*).

### Look for the single best tree

In this use case we are after just 1 tree. We specify up to what taxa size all possible trees for the sample will be computed. Here we are specifying a=5. This means 5 sequences will be picked randomly and all possible trees relating them will be evaluated. k=1 means only the best tree will be kept at the end of each such round of evaluation. For every remaining sequence it is grafted onto every possible branch of this tree. The best k results are then taken to the next round, when another sequence is randomly selected for addition. This proceeds until all sequences have been added. The result with following arguments is a single wls score and a single Tree which can be saved etc ..

```
>>> score, tree = ls.trex(a = 5, k = 1, show_progress = True)
3 trees of size 4 at start
15 trees of size 5 ... done
>>> print score
0.0009...
```

We won't display this tree, because we are doing more below.

### A more rigourous tree space search

We change the asaa settings, so we keep more trees and then look at the distribution of the statistics for the last collection of trees. We could also change a to be larger, but in the current case we just adjust k. We also set the argument return\_all = True, the effect of which is to return the complete set of 145 trees. These, and their support statistic, can then be inspected.

```
>>> trees = ls.trex(a = 5, k = 5, show_progress = True, return_all = True)
3 trees of size 4 at start
15 trees of size 5 ... done
```

Remember the sum-of-squares statistic will be smaller for 'good' trees. The order of the trees returned is from good to bad. As indicated above, trees has length 145.

```
>>> print len(trees)
15
```

Lets inspect the resulting statistics. First, the object trees is a list of (wls, Tree) tuples. We will therefore loop over the list to generate a separate list of just the wls statistics. The following syntax is called a list comprehension - basically just a very succinct for loop.

```
>>> wls_stats = [tree[0] for tree in trees]
>>> print wls_stats[:15]
[0.000914409229523, 0.001...
```

From this you'll see that the first 5 results are very similar to each other and would probably reasonably be considered equivalently supported topologies. I'll just print the first two of the these trees after balancing them (in order to make their representations as equal as possible).

```
>>> t1 = trees[0][1].balanced()
>>> t2 = trees[1][1].balanced()
>>> print t1.asciiArt()
          /-DogFaced
         |
         |          /-NineBande
-root----|-edge.0--|
         |          \-Mouse
         |
         |          /-HowlerMon
          \edge.1--|
                    \-Human
>>> print t2.asciiArt()
          /-NineBande
         |
         |          /-DogFaced
-root----|-edge.0--|
         |          \-Mouse
         |
         |          /-HowlerMon
          \edge.1--|
                    \-Human
```

You can see the difference involves the Jackrabbit, TreeShrew, Gorilla, Rat clade.

### Assessing the fit for a pre-specified tree topology

In some instances we may have a tree from the literature or elsewhere whose fit to the data we seek to evaluate. In this case I'm going load a tree as follows.

```
>>> from cogent import LoadTree
>>> query_tree = LoadTree(treestring = "((Human:.2,DogFaced:.2):.3,(NineBande:.1, Mouse:.5):.2,HowlerMon:.1)")
```

We now just use the ls object created above. The following evaluates the query using it's associated branch lengths, returning only the wls statistic.

```
>>> ls.evaluateTree(query_tree)
 3.95...
```

We can also evaluate just the tree's topology, returning both the wls statistic and the tree with best fit branch lengths.

```
>>> ls.evaluateTopology(query_tree)
(0.00316480233404, Tree("((Human,DogFaced),(NineBande,Mouse),HowlerMon)root;"))
```

### Using maximum likelihood for measuring tree fit

This is a much slower algorithm and the interface largely mirrors that for the above. The difference is you import maximum\_likelihood instead of least\_squares, and use the ML instead of WLS classes. The ML class requires a substitution model (like a HKY85 for DNA or JTT92 for protein), and an alignment. It also optionally takes a distance matrix, such as that used here, computed for the same sequences. These distances are then used to obtain estimates of branch lengths by the WLS method for each evaluated tree topology which are then used as starting values for the likelihood optimisation.

## Making a phylogenetic tree from a protein sequence alignment

In this example we pull together the distance calculation and tree building with the additional twist of using an empirical protein substitution matrix. We will therefore be computing the tree from a protein sequence alignment. We will first do the standard cogent import for LoadSeqs.

```
>>> from cogent import LoadSeqs, PROTEIN
```

We will use an empirical protein substitution matrix, this requires a file format parser also.

```
>>> from cogent.evolve.substitution_model import EmpiricalProteinMatrix
>>> from cogent.parse.paml_matrix import PamlMatrixParser
```

The next components we need are for computing the matrix of pairwise sequence distances and then for estimating a neighbour joining tree from those distances.

```
>>> from cogent.phylo import nj, distance
```

Now load our sequence alignment, explicitly setting the alphabet to be protein.

```
>>> aln = LoadSeqs('data/abglobin_aa.phylip', interleaved=True,
...                 moltype=PROTEIN)
```

We open the file that contains the empirical matrix and parse the matrix and frequencies.

```
>>> matrix_file = open('data/dayhoff.dat')
```

Create an Empirical Protein Matrix Substitution model object. This will take the unscaled empirical matrix and use it and the motif frequencies to create a scaled Q matrix.

```
>>> sm = EmpiricalProteinMatrix(*PamlMatrixParser(matrix_file))
```

We now use this and the alignment to construct a distance calculator.

```
>>> d = distance.EstimateDistances(aln, submodel = sm)
>>> d.run(show_progress=False)
```

The resulting distances are passed to the nj function.

```
>>> mytree = nj.nj(d.getPairwiseDistances())
```

The shape of the resulting tree can be readily view using the asciiArt method on Tree.

```
>>> print mytree.asciiArt()
          /-human
         |
         |          /-rabbit
-root----|-edge.1--|
         |          \-rat
         |
         |          /-goat-cow
          \edge.0--|
                    \-marsupial
```

This tree can be saved to file, the with\_distances argument specifies that branch lengths are to be included in the newick formatted output.

```
>>> mytree.writeToFile('test_nj.tree', with_distances=True)
```

# Python Coding Guidelines

## Why have coding guidelines?

As project size increases, consistency increases in importance. Unit testing and a consistent style are critical to having trusted code to integrate. Also, guesses about names and interfaces will be correct more often.

### What should I call my variables?

- *Choose the name that people will most likely guess.* Make it descriptive, but not too long: curr\_record is better than c, or curr, or current\_genbank\_record\_from\_database.
- *Good names are hard to find.* Don't be afraid to change names except when they are part of interfaces that other people are also using. It may take some time working with the code to come up with reasonable names for everything: if you have unit tests, it's easy to change them, especially with global search and replace.
- *Use singular names for individual things, plural names for collections.* For example, you'd expect self.Name to hold something like a single string, but self.Names to hold something that you could loop through like a list or dict. Sometimes the decision can be tricky: is self.Index an int holding a positon, or a dict holding records keyed by name for easy lookup? If you find yourself wondering these things, the name should probably be changed to avoid the problem: try self.Position or self.LookUp.
- *Don't make the type part of the name.* You might want to change the implementation later. Use Records rather than RecordDict or RecordList, etc. Don't use Hungarian Notation either (i.e. where you prefix the name with the type).
- *Make the name as precise as possible.* If the variable is the name of the input file, call it infile\_name, not input or file (which you shouldn't use anyway, since they're keywords), and not infile (because that looks like it should be a file object, not just its name).
- *Use* result *to store the value that will be returned from a method or function.* Use data for input in cases where the function or method acts on arbitrary data (e.g. sequence data, or a list of numbers, etc.) unless a more descriptive name is appropriate.
- *One-letter variable names should only occur in math functions or as loop iterators with limited scope.* Limited scope covers things like for k in keys: print k, where k survives only a line or two. Loop iterators should refer to the variable that they're looping through: for k in keys, i in items, or for key in keys, item in items. If the loop is long or there are several 1-letter variables active in the same scope, rename them.
- *Limit your use of abbreviations.* A few well-known abbreviations are OK, but you don't want to come back to your code in 6 months and have to figure out what sptxck2 is. It's worth it to spend the extra time typing species\_taxon\_check\_2, but that's still a horrible name: what's check number 1? Far better to go with something like taxon\_is\_species\_rank that needs no explanation, especially if the variable is only used once or twice.

#### Acceptable abbreviations

The following list of abbreviations can be considered well-known and used with impunity within mixed name variables, but some should not be used by themselves as they would conflict with common functions, python built-in's, or raise an exception. Do not use the following by themselves as variable names: dir, exp (a common math module function), in, max, and min. They can, however, be used as part of a name, eg matrix\_exp.

| Full | Abbreviated |
| --- | --- |
| alignment | aln |
| archaeal | arch |
| auxillary | aux |
| bacterial | bact |
| citation | cite |
| current | curr |
| database | db |
| dictionary | dict |
| directory | dir |
| end of file | eof |
| eukaryotic | euk |
| frequency | freq |
| expected | exp |
| index | idx |
| input | in |
| maximum | max |
| minimum | min |
| mitochondrial | mt |
| number | num |
| observed | obs |
| original | orig |
| output | out |
| parameter | param |
| phylogeny | phylo |
| previous | prev |
| probability | prob |
| protein | prot |
| record | rec |
| reference | ref |
| sequence | seq |
| standard deviation | stdev |
| statistics | stats |
| string | str |
| structure | struct |
| temporary | temp |
| taxonomic | tax |
| variance | var |

### What are the naming conventions?

| Type | Convention | Example |
| --- | --- | --- |
| function | verb\_with\_underscores | find\_all |
| variable | noun\_with\_underscores | curr\_index |
| constant | NOUN\_ALL\_CAPS | ALLOWED\_RNA\_PAIRS |
| class | MixedCaseNoun | RnaSequence |
| public property | MixedCaseNoun | IsPaired |
| private property | \_noun\_with\_leading\_underscore | \_is\_updated |
| public method | mixedCaseExceptFirstWordVerb | stripDegenerate |
| private method | \_verb\_with\_leading\_underscore | \_check\_if\_paired |
| really private data | \_\_two\_leading\_underscores | \_\_delegator\_object\_ref |
| parameters that match properties | SameAsProperty | def \_\_init\_\_(data, Alphabet=None) |
| factory function | MixedCase | InverseDict |
| module | lowercase\_with\_underscores | unit\_test |
| global variables | gMixedCaseWithLeadingG | no examples in evo - should be rare! |

- *It is important to follow the naming conventions because they make it much easier to guess what a name refers to*. In particular, it should be easy to guess what scope a name is defined in, what it refers to, whether it's OK to change its value, and whether its referent is callable. The following rules provide these distinctions.
- lowercase\_with\_underscores *for modules and internal variables (including function/method parameters).*
- MixedCase for *classes* and *public properties*, and for *factory functions* that act like additional constructors for a class.
- mixedCaseExceptFirstWord for *public methods and functions*.
- \_lowercase\_with\_leading\_underscore for *private* functions, methods, and properties.
- \_\_lowercase\_with\_two\_leading\_underscores for *private* properties and functions that *must not be overwritten* by a subclass.
- CAPS\_WITH\_UNDERSCORES for named *constants*.
- gMixedCase (i.e. mixed case prefixed with 'g') for *globals*. Globals should be used extremely rarely and with caution, even if you sneak them in using the Singleton pattern or some similar system.
- *Underscores can be left out if the words read OK run together.* infile and outfile rather than in\_file and out\_file; infile\_name and outfile\_name rather than in\_file\_name and out\_file\_name or infilename and outfilename (getting too long to read effortlessly).

### How do I organize my modules (source files)?

- *Have a docstring with a description of the module's functions*. If the description is long, the first line should be a short summary that makes sense on its own, separated from the rest by a newline.
- *All code, including import statements, should follow the docstring.* Otherwise, the docstring will not be recognized by the interpreter, and you will not have access to it in interactive sessions (i.e. through obj.\_\_doc\_\_) or when generating documentation with automated tools.
- *Import built-in modules first, followed by third-party modules, followed by any changes to the path and your own modules.* Especially, additions to the path and names of your modules are likely to change rapidly: keeping them in one place makes them easier to find.
- *Don't use* from module import \*, *instead use* from module import Name, Name2, Name3... *or possibly* import module. This makes it *much* easier to see name collisions and to replace implementations.

#### Example of module structure

```
#!/usr/bin/env python

"""Provides NumberList and FrequencyDistribution, classes for statistics.

NumberList holds a sequence of numbers, and defines several statistical
operations (mean, stdev, etc.) FrequencyDistribution holds a mapping from
items (not necessarily numbers) to counts, and defines operations such as
Shannon entropy and frequency normalization.
"""

from math import sqrt, log, e
from random import choice, random
from Utils import indices

class NumberList(list):
    pass    # much code deleted
class FrequencyDistribution(dict):
    pass    # much code deleted

# use the following when the module can meaningfully be called as a script.
if __name__ == '__main__':    # code to execute if called from command-line
    pass    # do nothing - code deleted
```

#### How should I write comments?

- *Always update the comments when the code changes.* Incorrect comments are far worse than no comments, since they are actively misleading.
- *Comments should say more than the code itself.* Examine your comments carefully: they may indicate that you'd be better off rewriting your code (especially, *renaming your variables* and getting rid of the comment.) In particular, don't scatter magic numbers and other constants that have to be explained through your code. It's far better to use variables whose names are self-documenting, especially if you use the same constant more than once. Also, think about making constants into class or instance data, since it's all too common for 'constants' to need to change or to be needed in several methods.

  > |  |  |
  > | --- | --- |
  > | Wrong | win\_size -= 20        # decrement win\_size by 20 |
  > | OK | win\_size -= 20        # leave space for the scroll bar |
  > | Right | self.\_scroll\_bar\_size = 20 |
  > |  | win\_size -= self.\_scroll\_bar\_size |
- *Use comments starting with #, not strings, inside blocks of code.* Python ignores real comments, but must allocate storage for strings (which can be a performance disaster inside an inner loop).
- *Start each method, class and function with a docstring using triple double quotes (""").* The docstring should start with a 1-line description that makes sense by itself (many automated formatting tools, and the IDE, use this). This should be followed by a blank line, followed by descriptions of the parameters (if any). Finally, add any more detailed information, such as a longer description, notes about the algorithm, detailed notes about the parameters, etc. If there is a usage example, it should appear at the end. Make sure any descriptions of parameters have the correct spelling, case, etc. For example:

  ```
  def __init__(self, data, name='', alphabet=None):
      """Returns new Sequence object with specified data, name, alphabet.

      Arguments:

          - data: The sequence data. Should be a sequence of characters.
          - name: Arbitrary label for the sequence. Should be string-like.
          - alphabet: Set of allowed characters. Should support 'for x in y'
            syntax. None by default.

      Note: if alphabet is None, performs no validation.
      """
  ```
- *Always update the docstring when the code changes.* Like outdated comments, outdated docstrings can waste a lot of time. "Correct examples are priceless, but incorrect examples are worse than worthless." Jim Fulton.

### How should I format my code?

- *Use 4 spaces for indentation.* Do not use tabs (set your editor to convert tabs to spaces). The behaviour of tabs is not predictable across platforms, and will cause syntax errors. If we all use the same indentation, collaboration is much easier.
- *Lines should not be longer than 79 characters.* Long lines are inconvenient in some editors. Use \ for line continuation. Note that there cannot be whitespace after the \.
- *Blank lines should be used to highlight class and method definitions.* Separate class definitions by two blank lines. Separate methods by one blank line.
- *Be consistent with the use of whitespace around operators.* Inconsistent whitespace makes it harder to see at a glance what is grouped together.

  > |  |  |
  > | --- | --- |
  > | Good | ((a+b)\*(c+d)) |
  > | OK | ((a + b) \* (c + d)) |
  > | Bad | ( (a+ b)  \*(c +d  )) |
- *Don't put whitespace after delimiters or inside slicing delimiters.* Whitespace here makes it harder to see what's associated.

  > |  |  |  |
  > | --- | --- | --- |
  > | Good | (a+b) | d[k] |
  > | Bad | ( a+b ) | d [k], d[ k] |

### How should I test my code ?

There are two basic approaches for testing code in python: unit testing and doc testing. Their purpose is the same, to check that execution of code given some input produces a specified output. The cases to which the two approaches lend themselves are different.

An excellent discourse on testing code and the pros and cons of these alternatives is provided in a presentation by Jim Fulton, which is recommended reading. A significant change since that presentation is that doctest can now read content that is not contained within docstrings. A another comparison of these two approaches, along with a third (py.test) is also available. To see examples of both styles of testing look in Cogent/tests: files ending in .rest are using doctest, those ending in .py are using unittest.

In general, it's easier to start writing doctest's, as you don't need to learn the unittest API but the latter give's much greater control.

Whatever approach is employed, the general principle is every line of code should be tested. It is critical that your code be fully tested before you draw conclusions from results it produces. For scientific work, bugs don't just mean unhappy users who you'll never actually meet: they may mean retracted publications.

Tests are an opportunity to invent the interface(s) you want. Write the test for a method before you write the method: often, this helps you figure out what you would want to call it and what parameters it should take. It's OK to write the tests a few methods at a time, and to change them as your ideas about the interface change. However, you shouldn't change them once you've told other people what the interface is.

Never treat prototypes as production code. It's fine to write prototype code without tests to try things out, but when you've figured out the algorithm and interfaces you must rewrite it *with tests* to consider it finished. Often, this helps you decide what interfaces and functionality you actually need and what you can get rid of.

"Code a little test a little". For production code, write a couple of tests, then a couple of methods, then a couple more tests, then a couple more methods, then maybe change some of the names or generalize some of the functionality. If you have a huge amount of code where 'all you have to do is write the tests', you're probably closer to 30% done than 90%. Testing vastly reduces the time spent debugging, since whatever went wrong has to be in the code you wrote since the last test suite. And remember to use python's interactive interpreter for quick checks of syntax and ideas.

Run the test suite when you change anything. Even if a change seems trivial, it will only take a couple of seconds to run the tests and then you'll be sure. This can eliminate long and frustrating debugging sessions where the change turned out to have been made long ago, but didn't seem significant at the time.

#### Some unittest pointers

- *Use the* unittest *framework with tests in a separate file for each module.* Name the test file test\_module\_name.py. Keeping the tests separate from the code reduces the temptation to change the tests when the code doesn't work, and makes it easy to verify that a completely new implementation presents the same interface (behaves the same) as the old.
- *Use* evo.unit\_test *if you are doing anything with floating point numbers or permutations* (use assertFloatEqual). Do *not* try to compare floating point numbers using assertEqual if you value your sanity. assertFloatEqualAbs and assertFloatEqualRel can specifically test for absolute and relative differences if the default behavior is not giving you what you want. Similarly, assertEqualItems, assertSameItems, etc. can be useful when testing permutations.
- *Test the interface of each class in your code by defining at least one* TestCase *with the name* ClassNameTests. This should contain tests for everything in the public interface.
- *If the class is complicated, you may want to define additional tests with names* ClassNameTests\_test\_type. These might subclass ClassNameTests in order to share setUp methods, etc.
- *Tests of private methods should be in a separate* TestCase *called* ClassNameTests\_private. Private methods may change if you change the implementation. It is not required that test cases for private methods pass when you change things (that's why they're private, after all), though it is often useful to have these tests for debugging.
- *Test `all` the methods in your class.* You should assume that any method you haven't tested has bugs. The convention for naming tests is test\_method\_name. Any leading and trailing underscores on the method name can be ignored for the purposes of the test; however, *all tests must start with the literal substring* test *for* unittest *to find them.* If the method is particularly complex, or has several discretely different cases you need to check, use test\_method\_name\_suffix, e.g. test\_init\_empty, test\_init\_single, test\_init\_wrong\_type, etc. for testing \_\_init\_\_.
- *Write good docstrings for all your test methods.* When you run the test with the -v command-line switch for verbose output, the docstring for each test will be printed along with ...OK or ...FAILED on a single line. It is thus important that your docstring is short and descriptive, and makes sense in this context.

  > **Good docstrings:**
  >
  > ```
  > NumberList.var should raise ValueError on empty or 1-item list
  > NumberList.var should match values from R if list has >2 items
  > NumberList.__init__ should raise error on values that fail float()
  > FrequencyDistribution.var should match corresponding NumberList var
  > ```
  >
  > **Bad docstrings:**
  >
  > ```
  > var should calculate variance           # lacks class name, not descriptive
  > Check initialization of a NumberList    # doesn't say what's expected
  > Tests of the NumberList initialization. # ditto
  > ```
- *Module-level functions should be tested in their own* TestCase*, called* modulenameTests. Even if these functions are simple, it's important to check that they work as advertised.
- *It is much more important to test several small cases that you can check by hand than a single large case that requires a calculator.* Don't trust spreadsheets for numerical calculations -- use R instead!
- *Make sure you test all the edge cases: what happens when the input is None, or '', or 0, or negative?* What happens at values that cause a conditional to go one way or the other? Does incorrect input raise the right exceptions? Can your code accept subclasses or superclasses of the types it expects? What happens with very large input?
- *To test permutations, check that the original and shuffled version are different, but that the sorted original and sorted shuffled version are the same.* Make sure that you get *different* permutations on repeated runs and when starting from different points.
- *To test random choices, figure out how many of each choice you expect in a large sample (say, 1000 or a million) using the binomial distribution or its normal approximation.* Run the test several times and check that you're within, say, 3 standard deviations of the mean.

#### Example of a unittest test module structure

```
#!/usr/bin/env python

"""Tests NumberList and FrequencyDistribution, classes for statistics."""

from unittest import TestCase, main # for floating point test use unittestfp
from statistics import NumberList, FrequencyDistribution

class NumberListTests(TestCase): # remember to subclass TestCase
    """Tests of the NumberList class."""
    def setUp(self):
        """Define a few standard NumberLists."""
        self.Null = NumberList()            # test empty init
        self.Empty = NumberList([])         # test init with empty sequence
        self.Single = NumberList([5])       # single item
        self.Zero = NumberList([0])         # single, False item
        self.Three = NumberList([1,2,3])    # multiple items
        self.ZeroMean = NumberList([1,-1])  # items nonzero, mean zero
        self.ZeroVar = NumberList([1,1,1])  # items nonzero, mean nonzero, variance zero
        # etc. These objects shared by all tests, and created new each time a method
        # starting with the string 'test' is called (i.e. the same object does not
        # persist between tests: rather, you get separate copies).

        def test_mean_empty(self):
            """NumberList.mean() should raise ValueError on empty object"""
            for empty in (self.Null, self.Empty):
                self.assertRaises(ValueError, empty.mean)
        def test_mean_single(self):
            """NumberList.mean() should return item if only 1 item in list"""
            for single in (self.Single, self.Zero):
                self.assertEqual(single.mean(), single[0])
        # other tests of mean
        def test_var_failures(self):
            """NumberList.var() should raise ZeroDivisionError if <2 items"""
            for small in (self.Null, self.Empty, self.Single, self.Zero):
                self.assertRaises(ZeroDivisionError, small.var)
        # other tests of var
        # tests of other methods

class FrequencyDistributionTests(TestCase):
    pass    # much code deleted
# tests of other classes

if __name__ == '__main__':    # run tests if called from command-line
    main()
```
