# Supplementary material for: Pitfalls of the most commonly used models of context dependent substitution
Source: Biol Direct. 2009 Mar 18;4:10. doi: 10.1186/1745-6150-4-10 (PMC2662811; doi:10.1186/1745-6150-4-10)
Supplement: Additional file 1 — Scripts used in the study. Archive of stand-alone web site presenting the central scripts used in this study. [file 1745-6150-4-10-S1.zip › HuttleyAdditional2/fit_dinuc_gtr.html]

Fitting the dinucleotide GTR model — Context Dependent Substitutions v-Draft documentation


### Navigation

- index
- next |
- previous |
- Context Dependent Substitutions v-Draft documentation »

# Fitting the dinucleotide GTR model¶

The general time reversible dinucleotide substitution model has 48 free parameters. Fitting this model is a challenge, with a tendency to obtaining a local optima. Consequently, we use an iterative fitting procedure – initially fitting the baseline model, then a model with an intermediate number of parameters followed by the full general time reversible dinucleotide model. In each case the simpler models are fit first and the resulting maximum likelihood estimators used as starting values for fitting the richer models. The substitution models were identically constructed, aside from the defining (matrix weighting) difference between the NF and TF forms, and optimised with the identical procedure.

The module responsible for executing this procedure (fit\_dinuc\_gtr.py) is shown below.

```
from __future__ import division
import os, optparse
from cogent import LoadSeqs, LoadTree, DNA, LoadTable
from cogent.evolve.substitution_model import Nucleotide

from fit_util import make_pwise, _nucs, _dinucs, opt,\
    motif_probs_from_monomers, get_ci, get_data_subpath, create_path

def make_mixed_nuc_di_preds():
    dinucs = make_pwise(_dinucs)
    nuc = make_pwise(_nucs)
    # for each nuc pred we eliminate one of the intersecting dinucs predicates after
    # eliminating one
    di_preds = []
    nuc.pop(-1)
    for np in nuc:
        nfr,nto = np.from_motif, np.to_motif
        preds = []
        for d in dinucs:
            diff = [[f, t] for f, t in zip(d.from_motif, d.to_motif) if f != t][0]
            if diff[0] == nfr and diff[1] == nto:
                preds.append(d)
        preds.pop()
        di_preds.extend(preds)
    return nuc + di_preds

def _param_matrix_val_dict(lf):
    lf_param_matrix = lf._model.getMatrixParams()
    lf_params = lf._model.getParamList()
    lf_params = [(n, lf.getParamValue(n)) for n in lf_params]
    return lf_param_matrix, dict(lf_params)

def _init_higher_model_params(poor, rich):
    """take a likelihood function of a less parameter rich substitution model,
    exract the param values, apply those to the corresponding GTR terms.
    
    Assumes rich has a single parameter in a cell and the param matrix is
    fully balanced."""
    poor_matrix, poor_params = _param_matrix_val_dict(poor)
    rich_matrix, rich_params = _param_matrix_val_dict(rich)
    dim = poor_matrix.shape[0]
    for i in range(dim-1):
        for j in range(i+1, dim):
            poor_cell = poor_matrix[i][j]
            if poor_cell:
                cell_value = 1.0
                for param in poor_cell:
                    cell_value *= poor_params[param]
                rich_param = rich_matrix[i][j]
                assert len(rich_param) == 1
                rich_param = rich_param[0]
                rich_params[rich_param] = cell_value
    for param, init in rich_params.items():
        init = min(1000, init)
        init = max(1e-6, init)
        rich.setParamRule(param, init=init)
    
    return rich

def fit_di_gtr(aln, use_monomer_probs, with_CI=False, show_progress=True, max_evaluations = 1000000, level=3):
    """returns a table object with collated statistics after incrementally
    fitting first a baseline dinucleotide model, then mixed nucleotide /
    dinucleotide model, followed by the Dinucleotide GTR model. In each case,
    parameter MLEs from the simpler model are used as initial values for the
    more complex model.
    
    Arguments:
        - aln: sequence alignment
        - use_monomer_probs: nucleotide freq form when True, Tuple freq form
          when False.
        - with_CI: whether to estimate parameter confidence intervals, default
          is False.
        - show_progress: optimiser progress
        - max_evaluations: maximum function evaluations for optimiser
        - level: 1=BASE, 2=Intermed, 3=Dinuc-GTR
    """
    # we fit the baseline model first
    if show_progress:
        print "\nFitting Dinuc nuc baseline"
    nuc_baseline = make_pwise(_nucs)
    nuc_baseline.pop(-1)
    submod_args = dict(mprob_model=[None,'monomer'][use_monomer_probs],
                        motif_length=2)
    di_mod = Nucleotide(predicates=nuc_baseline, **submod_args)
    tree = LoadTree(tip_names=aln.Names[:])
    null_lf = di_mod.makeLikelihoodFunction(tree)
    null_lf.setAlignment(aln)
    if not opt(null_lf, global_opt=True, show_progress=show_progress,
            max_evaluations=max_evaluations, model_name="Dinuc nuc baseline"):
        # we failed to optimise, so we exit
        return None
    
    if level == 1:
        table = get_ci(null_lf, table_name='BASE', with_motif_probs=True, with_CI=with_CI)
        table.Title=''
        return table
    
    if show_progress:
        print "Getting annotated tree 1"
    
    annot_tree = null_lf.getAnnotatedTree()
    
    # fit mixed dinucleotide and nucleotide terms
    if show_progress:
        print "\nFitting Mixed Nucleotide/Dinucleotide model"
    nt_di_preds = make_mixed_nuc_di_preds()
    di_mod = Nucleotide(predicates=nt_di_preds, **submod_args)
    null_lf = di_mod.makeLikelihoodFunction(annot_tree)
    null_lf.setAlignment(aln)
    if show_progress:
        print null_lf
        print null_lf.getLogLikelihood()
    
    if not opt(null_lf, global_opt=True, show_progress=show_progress,
            max_evaluations=max_evaluations, model_name="Mixed Nuc/Dinuc"):
        # we failed to optimise, so we exit
        return None
    
    if level == 1:
        table = get_ci(null_lf, table_name='Dinuc-intermed', with_motif_probs=True, with_CI=with_CI)
        table.Title=''
        return table
    
    null_lnL = null_lf.getLogLikelihood()
    null_lf.setName("Mixed Nuc/Dinuc model")
    
    if show_progress:
        print "Getting annotated tree 2"
    
    annot_tree = null_lf.getAnnotatedTree()
    
    if show_progress:
        print null_lf
    # make the dinucs GTR preds, where every possible dinucs exchange (-1) has its own param
    di_gtr_preds = make_pwise(_dinucs)
    di_gtr_preds.pop() # we delete one list entry
    di_gtr_mod = Nucleotide(predicates=di_gtr_preds, **submod_args)
    alt_lf = di_gtr_mod.makeLikelihoodFunction(annot_tree)
    alt_lf.setName("Dinuc GTR model")
    # set starting values for this function from the mixed nucleotide/dinucleotide
    # model
    alt_lf = _init_higher_model_params(null_lf, alt_lf)
    alt_lf.setAlignment(aln)
    if show_progress:
        print "\nFitting Dinucleotide GTR"
    
    if not opt(alt_lf, global_opt=True, show_progress=show_progress,
                    max_evaluations=max_evaluations, model_name="Dinuc GTR"):
        # we failed to optimise, so we exit
        return None
    
    table = get_ci(alt_lf, table_name='Dinuc-GTR', with_motif_probs=True, with_CI=with_CI)
    alt_lnL = alt_lf.getLogLikelihood()
    
    if alt_lnL < null_lnL:
        print "\nFAILED: Di GTR fit [%.4e] was worse than mixed model [%.4e]" % (alt_lnL, null_lnL)
        return None
    
    table.Title=''
    return table

def run(align_dir, begin, end, NF, overwrite, with_CI, level, test_run):
    """the dinuc GTR model is iteratively fit to individual alignments
    specified in the directory listing in the range begin-end. Results are
    written per alignment to matching the data/some_path/align_name as
    results/some_path/align_name.txt"""
    model_name = ['TF', 'NF'][NF]
    level_name={1: 'dinuc_base', 2: 'dinuc_intermed', 3: 'dinuc_gtr'}
    result_dir = os.path.join('../results', get_data_subpath(align_dir),
                              level_name[level], model_name)
    create_path(result_dir)
    fns = [fn for fn in os.listdir(align_dir) if fn.endswith('.fasta')]
    fns = fns[begin: end]
    for index, fn in enumerate(fns):
        print index, with_CI
        outfile_name = os.path.join(result_dir, fn.replace('.fasta', '.txt'))
        if not overwrite and os.path.exists(outfile_name):
            print outfile_name
            if test_run:
                print 'Already done'
            continue
        
        aln = LoadSeqs(os.path.join(align_dir, fn), moltype=DNA)
        result = fit_di_gtr(aln, NF, with_CI, test_run, level=level)
        
        if test_run:
            print result
            print outfile_name
            break
        
        result.writeToFile(outfile_name, sep="\t")
    
    
if __name__ == "__main__":
    parser = optparse.OptionParser()
    parser.add_option('--dirname', default=None)
    parser.add_option("--align_nums",default=None,help="alignment file range")
    parser.add_option('--NF', action='store_true', default=False,
        help='use the nucleotide frequency weighted model, default is False')
    parser.add_option('--level',type='int', default=3,
                    help='level of model fitting to exit')
    parser.add_option("--overwrite",action='store_true',default=False,
        help="overwrite existing results")
    parser.add_option("--with_CI", action='store_true',default=False,
        help="compute the confidence intervals")
    parser.add_option('--test_run', action='store_true', default=False)
    
    options, args = parser.parse_args()
    assert None not in (options.dirname,)
    b, e = map(int,options.align_nums.split('-'))
    
    run(options.dirname, b, e, options.NF, options.overwrite, options.with_CI,
            options.level, options.test_run)
    print "\n\nDone!"
```

This module depends on a utility module (fit\_util.py) whose functions are also employed for the fitting of individual dinucleotide parameters.

```
from __future__ import division
import subprocess, os

# following line to ensure using the correct version of PyCogent
from cogent.evolve import motif_prob_model

from cogent.evolve.predicate import replacement, MotifChange
from cogent import LoadTable, DNA

_dinucs = ["".join(list(pair)) for pair in list(DNA.Alphabet**2)]
_nucs = list(DNA)

def get_data_subpath(data_path):
    """returns the sup-path under data/"""
    split_path = data_path.split('/')
    sub_path = split_path[split_path.index('data')+1:]
    sub_path = os.path.join(*sub_path)
    return sub_path

def create_path(path):
    """creates path, raising an error"""
    if not os.path.exists(path):
        r = subprocess.call(["mkdir", "-p", path])
        if r != 0:
            raise RuntimeError, "Failed creating %s" % path
    

def one_diff(x, y):
    """returns True if motifs x and y differ at a single position"""
    diffs = [1 for i, j in zip(x,y) if i != j]
    return sum(diffs) == 1

def make_pwise(motifs):
    """makes predicates for all possible instantaneous pairwise exchanges
    among motifs"""
    preds = []
    for i in range(len(motifs)-1):
        for j in range(i+1,len(motifs)):
            x, y = motifs[i], motifs[j]
            if one_diff(x,y):
                preds.append(MotifChange(x,y))
    return preds

def motif_probs_from_monomers(motifs, monomer_probs):
    """returns the motif probabilities calculated as the product of
    the monomer probabilities"""
    if '-' in monomer_probs:
        # we remove count's of gap char, and normalise remainder to sum to 1
        monomer_probs.pop('-')
        total = sum(monomer_probs.values())
        for m,v in monomer_probs.items():
            monomer_probs[m] /= total
    
    motif_probs = {}
    for motif in motifs:
        v = 1.0
        for char in motif:
            v *= monomer_probs[char]
        motif_probs[motif] = v
    total = sum(motif_probs.values())
    for m,v in motif_probs.items():
        motif_probs[m] /= total
    return motif_probs

def display_lf(lf):
    """print lf without the motif probs"""
    print '\n'.join(map(str, lf.getStatistics(with_motif_probs=False,
                                with_titles=True)))

def get_ci(lf, with_motif_probs=True, table_name=None, with_CI=False):
    """returns a table with MLEs for parameters"""
    tables = lf.getStatistics(with_motif_probs=with_motif_probs,
                                with_titles=True)
    stats = []
    for table in tables:
        if 'global' in table.Title:
            for param in table.Header:
                try:
                    if with_CI:
                        stats += [("exchange", param) + \
                            lf.getParamInterval(param)]
                    else:
                        stats += [("exchange", param, '-',
                                    lf.getParamValue(param), '-')]
                except AssertionError:
                    continue
        
        elif 'edge' in table.Title:
            param_names = [n for n in table.Header[3:]]
            for param_name in param_names:
                param_sets = dict(table.getRawData([param_name, 'edge']))
                for edge in param_sets.values():
                    if 'omega' in param_name and with_CI:
                        row = [("exchange", '%s-%s' % (param_name, edge)) + \
                            lf.getParamInterval(param_name, edge)]
                    else:
                        row = [("exchange", '%s-%s' % (param_name, edge),
                                "-", lf.getParamValue(param_name, edge), "-")]
                    stats += row
            
        
        elif 'motif' in table.Title:
            stats += [("mprobs", m, "-", p, "-") for m,p in table.getRawData()]
    
    lnL = lf.getLogLikelihood()
    nfp = lf.getNumFreeParams()
    stats += [("lnL", "lnL", "-", lnL, "-")]
    stats += [("nfp", "nfp", "-", nfp, "-")]
    
    header = ["ParamType", "Name", "Lower", "MLE", "Upper"]
    table = LoadTable(header=header, rows=stats,
                      title=table_name or lf.getName())
    return table

def opt(lf, global_opt=False, show_progress=False, max_evaluations=100000, model_name=None):
    """function to standardise optimisation of likelihood functions and check
    they completed successfully"""
    if global_opt: # global optimiser first, then a local optimiser
        lf.optimise(local=False, show_progress=show_progress, tolerance=1.0)
    calc = lf.optimise(local=True, show_progress=show_progress, max_restarts=10,
                            return_calculator=True,
                            max_evaluations=max_evaluations)
    succeed = True
    if calc.evaluations > max_evaluations:
        if not model_name is None:
            print "FAILED (%d evals): %s was not sucessully optimised" % \
                                    (calc.evaluations, model_name)
        succeed = False
    
    return succeed
```

#### Previous topic

Filtering masked alignments

#### Next topic

Fitting individual dinucleotide parameters

### This Page

- Show Source

### Quick search

### Navigation

- index
- next |
- previous |
- Context Dependent Substitutions v-Draft documentation »

© Copyright 2008, Gavin Huttley.
Last updated on Dec 11, 2008.
Created using Sphinx 0.5.
