# Supplementary material for: Pitfalls of the most commonly used models of context dependent substitution
Source: Biol Direct. 2009 Mar 18;4:10. doi: 10.1186/1745-6150-4-10 (PMC2662811; doi:10.1186/1745-6150-4-10)
Supplement: Additional file 1 — Scripts used in the study. Archive of stand-alone web site presenting the central scripts used in this study. [file 1745-6150-4-10-S1.zip › HuttleyAdditional2/fit_dinuc_individ.html]

Fitting individual dinucleotide parameters — Context Dependent Substitutions v-Draft documentation


### Navigation

- index
- previous |
- Context Dependent Substitutions v-Draft documentation »

# Fitting individual dinucleotide parameters¶

We examined the consistency between statistics derived from the NF and TF model forms. Specifically, we contrast the likelihood ratio tests which compare a baseline and dinucleotide parameter against the simpler baseline parameterisation, and the maximum likelihood estimators obtained under the alternate hypotheses. The substitution models were identically constructed, aside from the defining difference between the NF and TF forms (matrix weighting), and optimised with the identical procedure.

The module responsible to executing this procedure (fit\_dinuc\_individ.py) is shown below.

```
from __future__ import division
import os, optparse
from pprint import pprint
from cogent import LoadSeqs, LoadTree, DNA, LoadTable
from cogent.evolve.substitution_model import Nucleotide

from fit_util import make_pwise, _nucs, _dinucs, opt,\
    motif_probs_from_monomers, get_ci, get_data_subpath, create_path

def iterate_specific_dinuc_preds(test_run=False):
    """returns the (name, pred) series
    
    pred series is a list of the nuc_GTR preds and one dinucleotide pred which
    can be used to directly specify a substitution model"""
    nuc_baseline = make_pwise(_nucs)
    nuc_baseline.pop(-1)
    # return the nucleotide baseline first
    yield 'baseline', nuc_baseline
    
    dinuc_gtr_preds = make_pwise(_dinucs)
    if test_run:
        dinuc_gtr_preds = dinuc_gtr_preds[:2]
    
    for dinuc in dinuc_gtr_preds:
        yield str(dinuc), nuc_baseline + [dinuc]

def fit_model_series(aln, use_monomer_probs, with_CI=False, show_progress=True, max_evaluations = 1000000):
    """fit's a series of different predicate sets to the same alignment. The
    BASE model (only nucleotide GTR predicates) is fit first. The MLEs from
    the BASE model are used for each subsequent variant: BASE+dinuc_param.
    
    Arguments:
         - aln: sequence alignment
         - use_monomer_probs: nucleotide freq form when True, Tuple freq form
           when False.
         - with_CI: whether to estimate parameter confidence intervals, default
           is False.
         - show_progress: optimiser progress
         - max_evaluations: maximum function evaluations for optimiser
    """
    assert len(aln.Names) == 3, "Designed for 3 OTUs only"
    annot_tree = None # will be created from the baseline model
    submod_args = dict(mprob_model='monomer', motif_length=2)
    # we make an unrooted tree of the triple
    tree = LoadTree(tip_names=aln.Names[:])
    count = 0
    tables = []
    is_first_model=True
    for i, (name, params) in enumerate(iterate_specific_dinuc_preds()):
        count += 1
        submod = Nucleotide(predicates=params, **submod_args)
        tr = [annot_tree, tree][annot_tree is None]
        lf = submod.makeLikelihoodFunction(tr)
        lf.setAlignment(aln)
        lf.setName(name)
        print "\nWorking on [%d]: %s" % (count, name)
        
        if not opt(lf, global_opt=is_first_model, show_progress=show_progress,
                        max_evaluations=max_evaluations):
            print 'FAIL: Not optimised'
            continue
        
        mle_table = get_ci(lf, table_name=name,
                           with_motif_probs=is_first_model, with_CI=with_CI)
        tables += [mle_table]
        
        is_first_model = False
        if annot_tree is None:
            annot_tree = lf.getAnnotatedTree()
    
    result = tables[0].appended('Model', tables[1:], title='')
    return result

def run(align_dir, begin, end, NF, overwrite, with_CI, test_run):
    """the BASE and BASE+dinuc_param model series are iteratively fit to
    individual alignments specified in the directory listing in the range
    begin-end. Results are written per alignment to matching the
    data/some_path/align_name as results/some_path/align_name.txt"""
    model_name = ['TF', 'NF'][NF]
    result_dir = os.path.join('../results', get_data_subpath(align_dir),
                              'dinuc_individual', model_name)
    create_path(result_dir)
    fns = [fn for fn in os.listdir(align_dir) if fn.endswith('.fasta')]
    fns = fns[begin: end]
    for index, fn in enumerate(fns):
        print index
        outfile_name = os.path.join(result_dir, fn.replace('.fasta', '.txt'))
        if not overwrite and os.path.exists(outfile_name):
            print outfile_name
            if test_run:
                print 'Already done'
            continue
        
        aln = LoadSeqs(os.path.join(align_dir, fn), moltype=DNA)
        result = fit_model_series(aln, NF, with_CI, test_run)
        
        if test_run:
            print result
            print outfile_name
            break
        
        result.writeToFile(outfile_name, sep="\t")
    

if __name__ == "__main__":
    parser = optparse.OptionParser()
    parser.add_option('--dirname', default=None)
    parser.add_option("--align_nums",default=None,help="alignment file range")
    parser.add_option('--NF', action='store_true', default=False,
        help='use the nucleotide frequency weighted model, default is False')
    parser.add_option("--overwrite",action='store_true',default=False,
        help="overwrite existing results")
    parser.add_option("--with_CI", action='store_true',default=False,
        help="compute the confidence intervals")
    parser.add_option('--test_run', action='store_true', default=False)
    
    options, args = parser.parse_args()
    assert None not in (options.dirname,)
    b, e = map(int,options.align_nums.split('-'))
    
    run(options.dirname, b, e, options.NF, options.overwrite, options.with_CI,
            options.test_run)
    print "\n\nDone!"
```

This module also depends on the utility module (fit\_util.py), see previous section for contents.

#### Previous topic

Fitting the dinucleotide GTR model

### This Page

- Show Source

### Quick search

### Navigation

- index
- previous |
- Context Dependent Substitutions v-Draft documentation »

© Copyright 2008, Gavin Huttley.
Last updated on Dec 11, 2008.
Created using Sphinx 0.5.
