# Supplementary material for: Pitfalls of the most commonly used models of context dependent substitution
Source: Biol Direct. 2009 Mar 18;4:10. doi: 10.1186/1745-6150-4-10 (PMC2662811; doi:10.1186/1745-6150-4-10)
Supplement: Additional file 1 — Scripts used in the study. Archive of stand-alone web site presenting the central scripts used in this study. [file 1745-6150-4-10-S1.zip › HuttleyAdditional2/search.html]

Search — Context Dependent Substitutions v-Draft documentation


### Navigation

- index
- Context Dependent Substitutions v-Draft documentation »

# Search

From here you can search these documents. Enter your search
words into the box below and click "search". Note that the search
function will automatically search for all of the words. Pages
containing fewer words won't appear in the result list.

### Navigation

- index
- Context Dependent Substitutions v-Draft documentation »

© Copyright 2008, Gavin Huttley.
Last updated on Dec 11, 2008.
Created using Sphinx 0.5.
