# Supplementary material for: Pitfalls of the most commonly used models of context dependent substitution
Source: Biol Direct. 2009 Mar 18;4:10. doi: 10.1186/1745-6150-4-10 (PMC2662811; doi:10.1186/1745-6150-4-10)
Supplement: Additional file 1 — Scripts used in the study. Archive of stand-alone web site presenting the central scripts used in this study. [file 1745-6150-4-10-S1.zip › HuttleyAdditional2/tf_affected_by_composition.html]

Parameters estimated from TF models are affected by composition — Context Dependent Substitutions v-Draft documentation


### Navigation

- index
- next |
- previous |
- Context Dependent Substitutions v-Draft documentation »

# Parameters estimated from TF models are affected by composition¶

We demonstrate this here by simulating under a standard nucleotide substitution model with asymmetric (AT-rich) nucleotide composition. We first import the necessary PyCogent components.

```
>>> from __future__ import division
>>> import os
>>> from cogent import LoadSeqs, LoadTree, LoadTable, DNA
>>> from cogent.evolve.predicate import MotifChange
>>> from cogent.evolve.substitution_model import Nucleotide
>>> from fit_util import opt
>>> from fit_dinuc_individ import iterate_specific_dinuc_preds
```

The tree was estimated from a single human alignment using the nucleotide general time reversible model (GTR). We first define a function for estimating parameters from a single alignment of primate introns.

```
>>> def get_simulator():
...     """first fits a BASE model of NF form to primate intron
...     alignment ENSG00000003147-0-50000.fasta, returns opt lf."""
...     in_path = '../data/primate_introns'
...     fn = 'ENSG00000003147-0-50000.fasta'
...     aln = LoadSeqs(os.path.join(in_path, fn), moltype=DNA)
...     tree = LoadTree(tip_names=['human', 'chimpanzee', 'macaque'])
...     sm=Nucleotide(predicates=BASE_PREDS, mprob_model='monomer',
...                   word_length=2)
...     lf = sm.makeLikelihoodFunction(tree)
...     lf.setAlignment(aln)
...     if not opt(lf, show_progress=False):
...         raise RuntimeError, 'failed to optimise'
...     return lf.simulateAlignment
```

We return a reference to the simulateAlignment method.

```
>>> tree = LoadTree(treestring='(human,chimpanzee,macaque)')
>>> sim_align = get_simulator()
>>> sequence_length=10000
```

Dinucleotide substitution models are specified by setting the word\_length argument equal to two. The switch between TF and NF weighted rate matrices is controlled by the mprob\_model argument. By default, the motif probabilities are estimated from the alignment. These will be dinucleotide probabilities in the case of the TF model, and nucleotide probabilities in the case of the NF model. The rate matrix is further modified by the specification of exchangeability parameters which govern the rate of exchange between sequence states. In PyCogent, these are referred to as model predicates. We further note here that the default value for parameters other than motif probabilities is 1.

We define a model predicate parameter for any substitution involving the CpG dinucleotide, ie CG<->NN. This predicate will be used to define both the tuple frequency (TF) and nucleotide frequency (NF) weighted models. (We note that the following statement is equivalent to MotifChange("CG", "NN").aliased('CG')).

```
>>> cg = MotifChange('CG').aliased('CG')
```

We now define a TF substitution model (which includes this CG<->NN predicate) and then likelihood function,

```
>>> tf_dinuc = Nucleotide(predicates = BASE_PREDS+[cg], word_length=2, mprob_model=None)
>>> tf_lf = tf_dinuc.makeLikelihoodFunction(tree)
```

and the NF compatible substitution model and likelihood function.

```
>>> nf_dinuc = Nucleotide(predicates = BASE_PREDS+[cg], word_length=2, mprob_model='monomer')
>>> nf_lf = nf_dinuc.makeLikelihoodFunction(tree)
```

We simulate 1000 alignments, optimise each model separately using the same optimisation profile (global optimiser followed by local optimiser) and record the parameter values for the CG<->NN parameter estimated under each form. The results are then written to a tab delimited file.

```
>>> num_reps = 1000
>>> rows = []
>>> for i in range(num_reps):
...     print i
...     aln = sim_align(sequence_length=sequence_length)
...     # We fit the TF model
...     tf_lf.setAlignment(aln)
...     tf_lf.optimise(max_restarts=5, show_progress=False)
...     # We fit the NF model
...     nf_lf.setAlignment(aln)
...     nf_lf.optimise(max_restarts=5, show_progress=False)
...     rows += [[nf_lf.getParamValue('CG'), tf_lf.getParamValue('CG')]]
>>> table = LoadTable(header=['NF', 'TF'], rows=rows)
>>> table.writeToFile('../results/sim_base/NF/affect_of_composition.txt', sep='\t')
```

#### Previous topic

Scripts: Context Dependent Substitution

#### Next topic

Filtering masked alignments

### This Page

- Show Source

### Quick search

### Navigation

- index
- next |
- previous |
- Context Dependent Substitutions v-Draft documentation »

© Copyright 2008, Gavin Huttley.
Last updated on Dec 11, 2008.
Created using Sphinx 0.5.
